# Advancements in CRISPR-based *in vivo* gene therapy for hemophilia

**DOI:** 10.3389/fgeed.2026.1924733

**Published:** 2026-09-10

**Authors:** Xiaoyu Zhang, Kaidi Xu, Shihui Zou, Zhiyao Zhu, Shuzhi Teng, Ping Huang

**Affiliations:** 1 The Key Laboratory of Pathobiology, Ministry of Education, Norman Bethune College of Medicine, Jilin University, Changchun, China; 2 The Third Norman Bethune Clinical College of Jilin University, Changchun, China; 3 Department of Internal Medicine, Changchun People’s Hospital, Changchun, China

**Keywords:** aav, CRISPR/Cas9, factor IX, factor VIII, gene editing, gene therapy, hemophilia, non-viral vectors

## Abstract

Hemophilia is an X-linked hereditary bleeding disorder caused by loss-of-function mutations in the genes encoding coagulation factors, leading to excessive bleeding and potentially being life-threatening. Currently, regular treatment for hemophilia is the infusion of recombinant blood coagulation factors. This approach is not only costly but can also give rise to complications such as the development of neutralizing antibodies (Nabs), which impede the therapeutic outcome. Hemophilia is a monogenic disease, making gene therapy a promising curative measure that includes gene addition and gene editing approaches. Adeno-associated virus (AAV) vectors have gained massive attention as the premier delivery vehicle for clinical gene therapy due to their diverse tissue tropisms dictated by the natural and engineered AAV capsids, their excellent safety profile since AAVs do not cause any known human diseases, low immunogenicity, and long-lasting gene expression. Several AAV-based gene addition therapies have been approved for hemophilia B and hemophilia A, and dozens of similar AAV-based clinical trials are underway. Gene editing technologies, like Clustered Regularly Interspaced Short Palindromic Repeats (CRISPR)/CRISPR-associated protein 9 (Cas9), have shown immense potential in the treatment of genetic diseases. CRISPR/Cas9-mediated transgene integration provides critical advantages over regular gene insertion therapy by enabling site-specific, targeted genomic integration rather than random integration. Clinical trials focusing on AAV-CRISPR/Cas9-mediated knock-in of human genes represent the frontier of *in vivo* genetic medicine, and one such prominent ongoing clinical trial for hemophilia B is designed to insert the human Factor IX gene into hepatocytes. This review outlines recent advances and feasible strategies of CRISPR/Cas9-based genome editing for hemophilia therapy, highlights the potential applications of next-generation gene editing and vector delivery technologies, and offers new insights for the advantages, limitations, and future directions for these novel treatment modalities.

## 1 Introduction

Hemophilia is a rare, inherited bleeding disorder that affects the clotting factors in the blood. The most common types are hemophilia A (HA) and hemophilia B (HB), both of which are X-linked recessive genetic disorders. The former results from a deficiency in blood clotting factor VIII (FVIII), while the latter is due to a deficiency in factor IX (FIX), each caused by mutations in their respective genes (Mannucci and Tuddenham, 2001).

Blood coagulation involves a series of clotting factors activated in a specific sequence. In other words, it is a cascade of proteolytic reactions. In each step, an inactive form of a clotting factor (a zymogen of a serine protease) and its glycoprotein cofactor are activated to become functional enzymes that subsequently catalyze the next reaction within the cascade (Smith et al., 2015). Different clotting factors are activated successively, ultimately leading to the formation and polymerization of fibrin (Supplementary Figure S1). Therefore, a deficiency in either FVIII or FIX will obstruct the normal clotting process, resulting in symptoms characterized by bleeding, primarily manifesting as joint bleeds and even intracranial or visceral bleeding (Berntorp et al., 2021). Based on the levels of FVIII or FIX in the blood, the severity of hemophilia can be classified into severe (<1% of normal), moderate (1%–5% of normal), and mild (>5%–40% of normal) (Berntorp et al., 2021). Spontaneous bleeding is common in severe cases, while patients with mild or moderate hemophilia may experience severe bleeding following injuries or surgeries. Inherited in an X-linked manner, the prevalences of hemophilia A and hemophilia B are approximately 1/5,000 and 1/30,000, respectively in the male population (Rodríguez-Merchán et al., 2021). According to the 2024 global survey by the World Federation of Hemophilia, the registered and total hemophilia patients are around 271,918 and 797,251, respectively, representing a major challenge for healthcare systems worldwide.

Currently, treatments for hemophilia include conventional recombinant clotting factor replacement therapy and non-factor prophylactic drugs, many of which are based on monoclonal antibodies such as emicizumab (Mahlangu et al., 2018). In the United States, recombinant blood clotting factors account for over 90% of the direct treatment costs for hemophilia, with the annual treatment cost reaching as high as $250,000 per adult patient (Rodriguez-Merchan, 2020). The FVIII products with standard half-life (SHL) and extended half-life (EHL) have half-lives of 10–12 h and 15–18 h, respectively (Berntorp et al., 2021). For FIX products, the SHL is 18–20 h, and the EHL is 2–5 times longer than SHL (Berntorp et al., 2021). Despite the existence of EHL products, repetitive infusions still cause discomfort and impose a burden on patients. Moreover, replacement therapy can lead to allergy and immune responses that result in the production of antibodies to the clotting factors, thereby impeding the normal clotting process and reducing the therapeutic efficacy. The cumulative incidence rate of this immune response is about 30% in patients with severe hemophilia A and about 3% in those with severe hemophilia B (Ragni, 2017). To overcome these limitations, new products have been developed to extend the half-life of recombinant clotting factors or to exert hemostatic effects through interfering with anticoagulant pathways, including using mutated clotting factors or mimics that can resist, suppress, or disrupt natural anticoagulants (Mast, 2016). Alternatively, subcutaneous administration of emicizumab, a bispecific monoclonal antibody that recognizes both FIX/FIXa and FX/FXa, mimics the cofactor function of FVIII by bridging FIX and its substrate FX and facilitates FIXa-mediated FX activation (Mahlangu et al., 2018). Despite the emergence of many new drugs, the necessity for regular and inconvenient injections remains a significant challenge for patients.

Hemophilia is a monogenic disease, making gene therapy a promising approach for a potential definitive cure. Clinical observations have demonstrated that even a minimal increase in clotting factor activity to >1% of the normal level significantly ameliorates the bleeding phenotype and enhances the quality of life (Gollomp et al., 2019). This makes hemophilia an ideal target for gene therapy because even moderate transgene expression/gene correction might confer significant benefits. Currently, there are two main approaches for hemophilia gene therapy. One is gene addition therapy, using gene delivery vectors (viral or non-viral) to directly transfer the normal clotting factor-encoding gene into the targeted cells (Samelson-Jones and George, 2023). The other approach, gene editing therapy, uses gene editing technologies like zinc-finger nucleases (ZFNs), transcription activator-like effector nucleases (TALENs), and CRISPR/Cas9-based systems to mediate the insertion of therapeutic genes or to precisely correct mutations *in situ* (Li et al., 2011; Park et al., 2014; Chen et al., 2019).

AAV-mediated gene addition therapy is a widely used approach, in which the functional *F8* or *F9* gene (or their optimized variants) is packaged into AAV and administered to the patient, resulting in episomal expression of the transgene (Samelson-Jones and George, 2023). Recombinant adeno-associated viruses (rAAV) mainly persist as circular concatemer episomes, which enable long-term transgene expression *in vivo*. The integration rate was low, with an average frequency of 0.31 and 0.07 in nonhuman primates and patients’ liver biopsies, respectively (Gil-Farina et al., 2016). However, high-dose AAV vector-induced insertion mutations were associated with the development of hepatocellular carcinoma in mice (Donsante et al., 2007). Fortunately, no associations have been found between AAV and the incidence of hepatocellular carcinoma in dogs or clinical trial participants (Leebeek and Miesbach, 2021; Batty et al., 2024). Moreover, over a decade of follow-up studies of treated hemophilia A dogs indicated that AAV-cFVIII vectors predominantly persist in the liver in nonintegrated episomal forms (Batty et al., 2024).

The first clinical trial for hemophilia gene therapy via intramuscular delivery of AAV2-FIX was launched in 1999, but multiple obstacles—AAV capsid-mediated immune responses, fluctuations in transgenic expression, and long-term safety concerns—led to approval delays of over 20 years (Herzog et al., 2025). In November 2022, the first gene therapy product for hemophilia B known as etranacogene dezaparvovec-drlb (Hemgenix) was approved (Pipe et al., 2023), which utilizes hepatic AAV5 to introduce the *F9 Padua* variant (R338L) and exhibits an eightfold increase in specific activity compared to wild type (WT) *F9* (Simioni et al., 2009). In 2023, the first hemophilia A gene therapy product, Roctavian (valoctocogene roxaparvovec-rvox), was approved in the United States as well, which operates on AAV5-based *F8* variant expression in hepatocytes (Samelson-Jones et al., 2024). Although promising in treating hemophilia, gene augmentation therapy is limited by transient transgene expression and potential immune responses to AAV vectors, both of which could lower its efficacy (Hurlbut et al., 2010; Chen et al., 2019). As for Roctavian treatment, progressive declines in FVIII expression were observed after the first 6–12 months: expression levels fell by around 40% 2 years after infusion, and no stable expression plateau was detected during 6-year follow-up. Moreover, high-titer, multiserotype cross-reactive AAV antibodies were generated, preventing repetitive AAV treatment (Samelson-Jones et al., 2024). This product was voluntarily withdrawn from the market in 2026, and the potential pitfalls of universal immune barriers with AAV offer vital design references for AAV-CRISPR platforms, guiding vector engineering, dose setting, and immune intervention schemes.

It is important to note that lentiviral vector (LVV)-mediated gene transfer is also applicable for gene insertion. In one study, autologous CD34^+^ hematopoietic stem cells from patients with severe hemophilia A were transduced with a lentiviral vector encoding CD68-ET3 (a novel *F8* transgene driven by a myeloid-directed CD68 promoter), achieving sustainable therapeutic levels of plasma FVIII expression and eliminating spontaneous bleeding without unexpected safety issues (Srivastava et al., 2025). In another clinical trial, a severe hemophilia A patient with FVIII inhibitor history was recruited, and *BDD-F8* driven by an ITGA2B (integrin subunit alpha 2b) promoter for platelet expression was delivered to autologous CD34^+^ peripheral blood stem cells via LVV. The patient exhibited steady platelet-derived FVIII expression, discontinued emicizumab 3.6 months after infusion, and was devoid of bleeding during a 24-month follow-up period (Eapen et al., 2025). These two phase I clinical trials suggest a new therapeutic option for hemophilia A, even though more participants and longer follow-up are necessary for verification of the safety and efficacy of LVV-mediated gene insertion.

Gene editing approaches, mediated by specific enzymatic systems, enable permanent modification of the target gene in the host genome, and offer a more durable and potentially curative solution for hemophilia. Gene editing is accomplished by engineered endonucleases that cut DNA at specific, targeted sites within a genome. The resulting DSB initiates the cell’s natural repair machinery, allowing insertion, deletion, or modification of specific gene sequences. ZFNs and TALENs utilize protein-based DNA-binding domains to target and cleave specific sequences for genomic DNA editing, but the requirement of engineering unique proteins for each target sequence makes the design process intricate and laborious (Randhawa and Sengar, 2021). In contrast, the CRISPR/Cas9 system relies on a single-guide RNA (sgRNA) to direct DNA targeting, which is simpler and more cost-effective. Its simplicity coupled with high efficiency and precision has made CRISPR-based systems the preferred tools for gene editing in hemophilia therapy. In this review we summarize different genome editing strategies for hemophilia therapy based on the CRISPR/Cas9 system, discuss the potential applications of emerging gene editing and vector delivery tools, and offer new insights for the gene editing treatment of hemophilia.

## 2 CRISPR/Cas9 for hemophilia treatment

**FIGURE 1:**
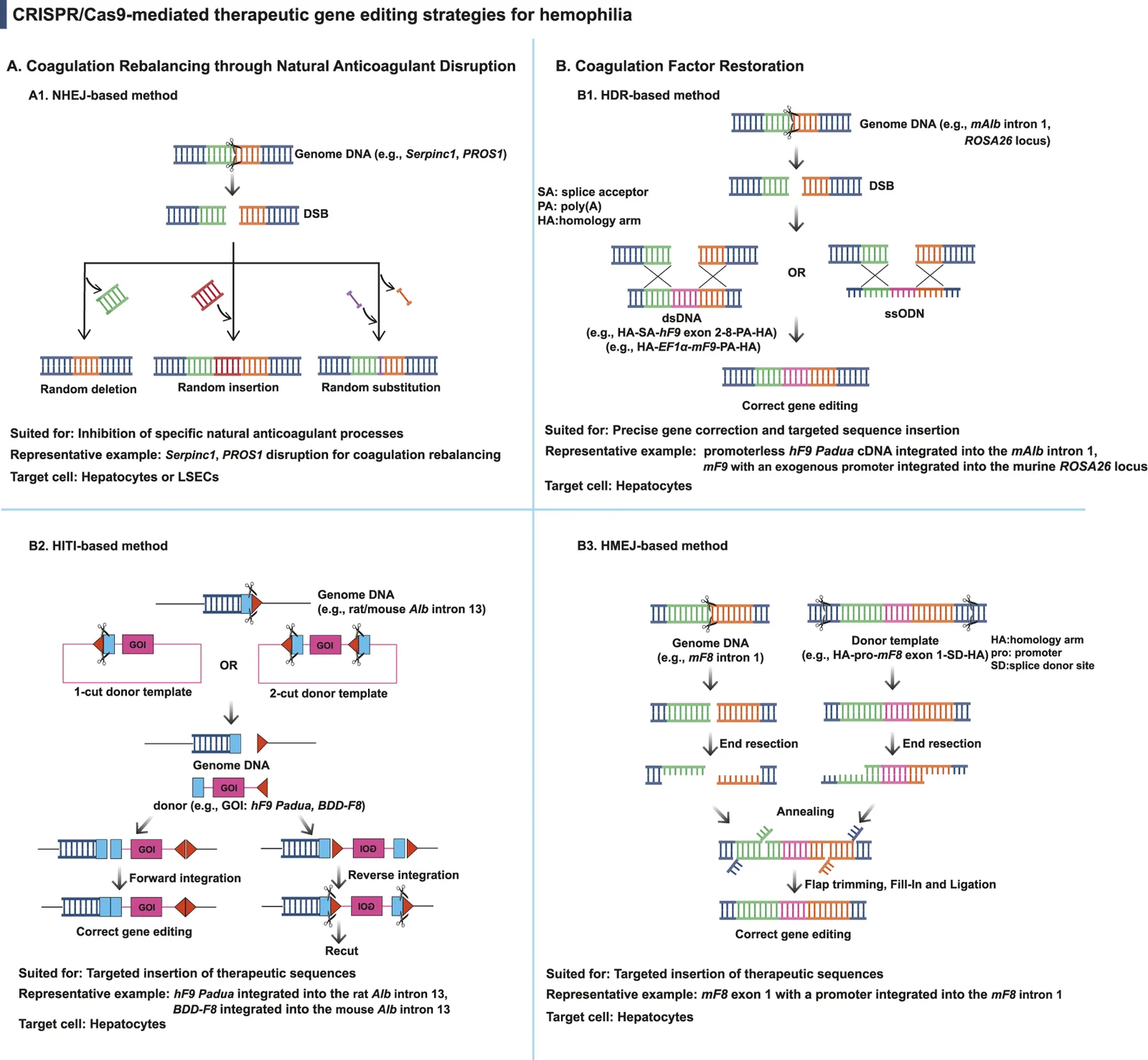
Overview of the CRISPR/Cas9-mediated gene editing for hemophilia therapy. In CRISPR/Cas9-mediated gene editing, Cas9 is directed to specific DNA sequences by sgRNA, causing a DSB at the target locus. **(A)** The DSB is repaired mainly via NHEJ, which occurs in all phases of the cell cycle with high efficiency but is poor in accuracy and may lead to random deletion, insertion, and substitution. **(B)** By introducing a donor DNA template, either dsDNA or ssODN, precise gene editing can be accomplished at the DSB site through HDR or single-stranded template repair, respectively. HDR only occurs in the S and G2 phases of the cell cycle, resulting in a lower repair frequency. **(B2)** For HITI-based gene knock-in, the donor DNA includes the gene of interest (GOI) and the same Cas9/sgRNA recognition sequences for the target genome but in the reverse direction. DSBs are generated simultaneously in both genomic DNA and donor DNA, ensuring donor integration into the genomic target site. If the donor DNA is integrated in the correct direction, sgRNA target sites are destroyed, whereas reverse integration will result in Cas9 recutting the donor template. **(B3)** The HMEJ-mediated gene knock-in incorporated the features of HDR and HITI, and the donor template for HMEJ includes integrating DNA, two sgRNA target sites, and two homology arms (∼800 bp) identical to the target genome. Homologous recombination (not shown) and HMEJ mechanisms may be involved in this method.

The CRISPR/Cas system is a natural immune mechanism in bacteria to combat phage infections (Garneau et al., 2010). Specifically, the CRISPR/Cas system precisely cleaves phage DNA at specific sites next to the protospacer adjacent motif (PAM), thus disrupting the genetic material of bacteriophages (Garneau et al., 2010). In CRISPR/Cas9-mediated gene editing, Cas9 is directed to specific DNA sequences by sgRNA, causing a double-strand break (DSB) at the target locus. The cell repairs this DSB via non-homologous end joining (NHEJ, Figure 1A) or microhomology-mediated end joining (MMEJ), leading to various insertions and deletions (indels) (Lieber, 2010; van Overbeek et al., 2016). Additionally, by introducing a donor DNA template homologous to the target site, precise gene editing can be accomplished at the DSB locus, with the donor sequences incorporated at the DSB site through homology-directed repair (HDR, Figure 1B) (Chapman et al., 2012).

Currently, research on hemophilia therapies using the CRISPR/Cas9 system is still in the preclinical phase. There are three major gene-editing strategies: (1) embedding the gene that encodes the missing or defective blood clotting factor at the specific site within the target genome, thus enabling the re-expression of the deficient blood clotting factor; (2) precisely repairing genetic mutations *in situ*, suitable for point mutations as well as small fragment insertions or deletions; (3) suppressing specific natural anticoagulant processes and restoring bleeding-associated phenotypes (Sehgal et al., 2015; Sharma et al., 2015; Guan et al., 2016).

### 2.1 CRISPR/Cas9-mediated therapeutic gene insertion for hemophilia treatment

Therapeutic transgenes are generally inserted in the genomic safe harbor (GSH) sites or the specific blood clotting factor gene location. The GSH site is a specific, relatively stable region of the genome where exogenous genes can be inserted without significantly affecting the host organism’s normal gene expression and function during gene editing and modification (Papapetrou and Schambach, 2016). In hemophilia gene therapy, the albumin (*Alb*) gene serves as a common GSH for therapeutic transgene insertion because albumin is highly expressed in hepatocytes, and transgene expression can be achieved by inserting a promoterless cassette containing the therapeutic gene together with a splice acceptor (SA) into *Alb* intron 1 (Sharma et al., 2015). This strategy offers a universal method to express and secrete proteins by leveraging albumin’s strong promoter and its first exon, which encodes a secretory peptide (Sharma et al., 2015). To avoid disruption of the normal albumin production, a bicistronic expression construct is employed for co-expression of the therapeutic protein and albumin.

Insertion of the normal *F8* or *F9* gene right after its endogenous promoter can re-express the specific blood clotting factor absent due to a gene mutation. Liver sinusoidal endothelial cells (LSECs) are believed to be the primary source of FVIII production (Fahs et al., 2014), while FIX is produced and secreted by hepatocytes. Due to limited types of delivery vectors designed to target LSECs, ectopic expression of the *F8* gene in GSH is more appropriate for HA treatment. Gene insertion at either a GSH site or the endogenous *F9* gene locus is suitable for HB treatment. Studies on CRISPR/Cas9-mediated *in vivo* targeted gene insertion for hemophilia are listed in Table 1.

**TABLE 1:** Studies on CRISPR/Cas9-mediated *in vivo* targeted gene insertion for hemophilia.

Disease	Transgene	Target site	Capsid	Ratio	Total dose	Editing efficiency	References
HA	*BDD-F8*	*mAlb* Intron11 or Intron 13	AAV8	(SpCas9): (sgRNA): (*BDD-F8*) = 1:1:5	7E11 GC/mouse (adult)	1%–2%	(Zhang et al., 2019)
HA	*BDD-F8* (with SQ/N6 sequence and R1645H)	*mAlb* Intron 13	AAV8	(SaCas9-sgRNA): (*BDD-F8*) = 1:5	2.4E10, 1.2E11 6E11 or 3E12 vg/kg (adult)	0.2%–0.3%	(Chen et al., 2019)
HA	promoter*- mF8 Exon 1*	*mF8* Intron 1	AAV8	(donor): (CRISPR/SaCas9) = 1:2	6E11 GC/mouse (adult) 1.2E11/mouse (low dose, adult)	NA	(Luo et al., 2021)
HA	*BDD-F8*	*Serpinc1*	AAV8	1E12 vg/kg of AAV donor and 1.5 mpk of LNP-CRISPR		0.13%	(Han et al., 2023)
HA	*BDD-F8*	*mAlb* Intron 13	AAV8	(SpCas9): (HITI-donor) = 1:1	3.9E13 GC/kg (neonate)	NA	(Esposito et al., 2024)
HA	*BDD-F8*	*mAlb Intron 13*	AAV8-*BDD-F8*:2E+12 vg/kg; LNP-SpCas9-sgRNA: 1 mg/kg			NA	(Zhao et al., 2025)
HB	*mF9* Exon 2–8	*mF9* Intron 1	AAV8	(SaCas9-sgRNA)+(*mF9* Exon 2–8)	1E12 (vg)+3E12 vg (high dose, adult) 0.3E12 vg+1E12 vg (low dose, adult) (6E10+2E11) vg (P0) (1.4–2.2E11 + 4.1–6.6E11) vg (P7) (1E12+3E12) vg (P28 and P42)	NA	(Ohmori et al., 2017)
HB	*mF9*	*ROSA26*	Ad5	(*EF1α*-*mF9*): (Cas9-sgRNA) = 3:1	1E11 VP/mouse (4 weeks old)	NA	(Stephens et al., 2019)
HB	*hF9 Padua*	*mF9* Exon 2	AAV8	(SaCas9): (sgRNA-*hF9co-Padua*) = 1:5 (P2) (SaCas9): (sgRNA-*hF9co-Padua*) = 1:10 (adult)	3E11 GC (P2) 5.5E12 GC (adult)	11% (P2) 3% and 9% (adult)	(Wang et al., 2019b)
HB	*hF9 Padua* Exon 2–8	*mAlb* Intron 1	AAV8	(SpCas9): (sgRNA-donor) = 1:5	1.8E11 GC/mouse (neonate) 5.4E11 GC/mouse (adult)	neonatal: 8.2%–16.1% at week 14 and 8.9%–15.9% at week 48 adult: 3.8% ± 0.8%	(Wang et al., 2020)
HB	*hF9* (carrying V86A, E277A, and R338L-Padua)	*mAlb* Exon 14	AAV8	(rAAV8-SaCas9): (rAAV8-donor-*hF9*) = 1:8 (P2) (rAAV8-SaCas9): (rAAV8-donor-*hF9*) = 1:5 (adult)	5.6E11 vg/mouse (neonate) 6E11 vg/mouse (adult)	4.02% (neonate) 0.35% (adult)	(Lisjak et al., 2022)
HB	*hF9 Padua*	*mAlb* Exon 14	AAV2/8	(IRES- *hF9 Padua*): (LP1-SpCas9): (sgRNA) = 2:1:1	2E9 vg/mouse (neonate) 1E10 vg/mouse (adult)	0.077% (neonate) 0.49% (adult)	(He et al., 2022)
HB	*hF9 Padua*	*rAlb* Intron 13	AAV8	(LP1-spCas9): (HITI-donor) = 1:2	6E11 GC/rat (low dose, adult) 6E12 GC/rat (high dose, adult)	0.52%–1.14% (low dose) 1.31%–3.66% (high dose)	(Chen et al., 2022b)
HB	*hF9*	*APOC3*	AAV8	(AAVs-TS-APOC3-CjCas9): (AAV-donor) = 1:1	2E13 vg/kg	NA	(Lee et al., 2022)
HB	*hF9*	*Serpinc1*	AAV8	2E13 vg/kg of AAV donor and 1.2 mpk of LNP-CRISPR		3%	(Lee et al., 2023)
HB	*mF7*	*ROSA26*	(AAV8-gRNA): (AAV_2_K66_5_Q-Cas9): (AAV8-mFVIIa) = 1:1:2		4E11 vg/mouse (neonate)	NA	(Sarangi et al., 2024)

*BDD-F8*, B domain deleted-F8; *hF9*, human *F9*; *mF9*, mouse *F9*; *mF8*, mouse *F8; mF7*, mouse *F7*; F9-Padua, F9-R338L; *rAlb*, rat *Alb*; mFVIIa, mouse activated FVII; vg, vector genomes; GC, genome copies; VP, viral particles; mpk, milligrams per kilogram; *APOC3*, apolipoprotein C3; *Serpinc1*, Serpin Family C Member 1; NA, not applicable.

Recent studies have shown that some variants of FVIII or FIX possess enhanced activity and can be expressed and secreted more efficiently. Thus, integrating *F8 or F9* variants into the genome can enhance therapeutic outcomes. FVIII contains a domain structure of A1-A2-B-A3-C1-C2, in which the B domain is non-essential for its coagulation activity (Toole et al., 1984). In fact, the B domain deleted-*F8* (*BDD-F8*) gene expression level is over 10-fold higher than that of the full-length *F8*, exhibiting increased FVIII secretion and significant therapeutic effects in animal models (Pittman et al., 1993). Since the full-length *F8* cDNA (∼7 kb) exceeds the packaging size (<5 kb) of the recombinant AAV (rAAV) vectors, variants of human *BDD-F8* with the B domain either completely or partially deleted are widely investigated in gene therapies for HA instead, including partial *BDD-F8* containing modified SQ and N6 sequences. The SQ sequence is a 14-amino acid linker formed by fusing Ser743 and Gln1638 in the B domain, which induces intracellular cleavage efficiently by containing the protease recognition site RHQR (Ward et al., 2011). The N6 sequence retains an N-terminal 226-amino acid B domain segment with six N-linked glycosylation sites known to be essential for ER-Golgi transport of FVIII (Ward et al., 2011). Additionally, an amino acid substitution at the furin cleavage site within the B domain (R1645H) was introduced to mimic the canine sequence (Siner et al., 2013). All these modifications have been shown to increase the secretion and activity of FVIII significantly in HA mice (Ward et al., 2011; Siner et al., 2013). In search of FIX variants with enhanced specific clotting activity, Kao et al. incorporated the FIX Padua mutation (R338L) (Simioni et al., 2009) into the FIX-Triple variant (V86A/E277A/R338A) (Lin et al., 2010) to produce FIX-TripleL (V86A/E277A/R338L), which demonstrated activity superior to any other variant (Kao et al., 2013). The FIX-TripleL possessed a 15-fold higher specific clotting activity than FIX-WT, compared to FIX-Triple (10-fold higher) and FIX-Padua (6-fold higher) in HB mice (Kao et al., 2013).

Previously Barzel et al. successfully integrated a promoterless human *F9* (*hF9*), along with the 2A peptide sequence, into the last exon of mouse *Alb* (*mAlb*) by appending homology arms to both ends of the *hF9* sequence without nucleases, resulting in *hF9* and *mAlb* expression simultaneously (Barzel et al., 2015). This so-called GeneRide technique was combined with CRISPR/SaCas9 (*Staphylococcus aureus* Cas9) to further increase the recombination rate and therapeutic efficacy (Lisjak et al., 2022). One-month post-intravenous injection of rAAV8-donor-hFIX-TripleL and AAV-SaCas9 vectors into neonatal *F9* knockout (KO) mice, plasma human FIX (hFIX) levels reached normal values, and by 10 months, they reached approximately 10,000 ng/mL (more than 200% of normal hFIX levels). Accordingly, the clotting capacity of treated *F9* KO mice was indistinguishable from that of WT mice. However, this treatment was less effective in adult hemophilic mice, as plasma hFIX was only ∼1% of the normal value, approximately 150 times lower than that observed in neonates at the same 2-month timepoint. Further analysis showed an average gene-targeting rate of 4.02% for the juvenile group and 0.35% for the adult group, and such low targeting rates in adult mice may be due to NHEJ instead of HDR serving as the predominant repair pathway in post-mitotic liver (Lisjak et al., 2022). This study also revealed that *hF9* mRNA levels were much higher than protein levels in both neonate and adult-treated mice, possibly due to inefficient protein translation of the *hF9* ORF in the chimeric *Alb*-*hF9* mRNA or an imbalance in the expression of the second ORF by the 2A peptide (Barzel et al., 2015).

In a similar study, Wang et al. treated HB mice by integrating the promoterless *hF9 Padua* cDNA (spanning exons 2–8) into the *mAlb* intron 1 via the HDR pathway, ensuring robust hFIX production and secretion under the control of the *mAlb* promoter (Wang et al., 2020). In adult HB mice, hFIX levels stabilized at 40% of normal levels 16 weeks after treatment, with activities reaching 162% ± 29% of normal. For neonatal HB mice, plasma hFIX levels remained consistent at 120% of normal values 4 weeks after treatment, with activities remarkably increasing to 590% ± 61%. This treatment strategy recovered the coagulation function of both adult and neonatal HB mice without significant off-target effects and affected Alb protein expression. Nevertheless, the high indel frequency (21%–34%) deserves attention, as it may lead to other adverse outcomes.

Differing from gene insertion at the *Alb* locus, incorporation of a gene at the safe harbor *ROSA26* site requires the inclusion of both the therapeutic transgene and an external promoter. Stephens et al. utilized adenovirus 5 to deliver the Cas9-sgRNA and EF1α-*mF9* (mouse *F9* with the exogenous EF1α promoter) into the *ROSA26* locus in HB mice (Stephens et al., 2019). One week after a single tail vein co-injection, the plasma FIX levels of all treated HB mice were within the ‘mild’ hemophilia range (5%–50% of normal FIX levels). By day 238 after injection, the Ad5-EF1α-*mF9* knock-in (KI) group maintained an average FIX level of 419 ng/mL and ∼5% of normal FIX activity, compared to the control group’s 88.5 ng/mL FIX level and ∼1% of normal FIX activity. This highlights that the integrated expression of the therapeutic transgene provides sustainable higher mFIX levels with superior long-term phenotypic correction as compared to the non-integrated group. However, targeted integration also elicited error-containing insertions and adaptive immune responses against the Cas9 protein and adenovirus in this study, necessitating further investigation.

As previously mentioned, the NHEJ pathway is more efficient than HDR to repair DSBs (Lisjak et al., 2022). Zhang et al. found that even when using the *BDD-F8* donor template with homology arms for targeting the *Alb* locus, gene insertion mediated by NHEJ was still more efficient than HDR (Zhang et al., 2019). Furthermore, using a double-cut donor (flanked by sgRNA-PAM sequences and released by Cas9 cleavage) instead of a traditional circular plasmid donor could enhance HDR editing efficiency by 10–20 times and lead to a complete reconstitution of FVIII coagulant activity 1 week after hydrodynamic tail vein (HTV) injection of CRISPR plasmids or AAV-CRISPR-BDDF8 therapy in HA mice (Zhang et al., 2019). Although promising, long-term follow-up of AAV-CRISPR-treated mice is essential. A comprehensive study on its safety is needed, such as monitoring problems associated with off-target editing, insertions of AAV-Cas9 sequences at the cleavage site, reverse insertion of *BDD-F8*, and reduced plasma albumin levels after treatment.

To enhance the likelihood of insertion of the donor template in the right direction via the NHEJ pathway, Chen et al. employed an NHEJ-based homology-independent targeted integration (HITI) strategy in which the *hF9 Padua* template was flanked at its 5′and 3′ends by the same Cas9/sgRNA recognition sequence of the endogenous target site (rat *Alb* intron 13) but in an inverted orientation (Chen X. et al., 2022). Upon simultaneous cleavage of the target sites of the genome and donor vector, should the linearized donor DNA integrate in the reverse direction at the cleavage site, the Cas9 cutting site is reassembled, and secondary cleavage occurs to remove the inserted sequence (Figure 1B2). Conversely, if the donor DNA is inserted in the correct orientation, Cas9 would not perform a second cut due to loss of target sites (Suzuki et al., 2016). The knock-in efficiency with this HITI strategy via rAAV8 delivery reached 3.66%, the insertion efficiency of the *hF9 Padua* cDNA in the correct direction was 7.5 times higher than that in the reverse direction, and the coagulation function was recovered after 4 weeks of treatment without significant off-target effects in an HB rat model (Chen X. et al., 2022). Following a similar strategy, Esposito et al. employed the HITI approach to insert *BDD-F8* into the *mAlb* intron 13 of HA mice (Esposito et al., 2024). In neonatal HA mice, AAV-SpCas9 (*Streptococcus pyogenes* Cas9) and AAV-HITI vectors were administered systemically at postnatal days 1–2, and plasma FVIII levels were restored to approximately 40% of WT levels at 90 days post-treatment. In adult HA mice, FVIII levels reached approximately 36% of normal levels 30 days after HITI treatment. Long-term safety assessments indicated that AAV-HITI does not impair albumin expression, nor does it induce hepatocellular carcinoma or significant chromosomal aberrations, supporting its potential clinical translation.

To further reduce the occurrence of indels and toxicity risks, He et al. substantially reduced the AAV vector doses (10- to 100-fold) when delivering the SpCas9, sgRNAs, and the *hF9 Padua* donor for the NHEJ-mediated KI of *hF9* to the *mAlb* 3′untranslated region (He et al., 2022). With a dose as low as 1 × 10^10^ vector genomes (vg)/mouse of AAV mixture for adult HB mice, effective hemostatic correction was achieved, yielding plasma hFIX Padua levels of approximately 350 ng/mL and nearing 30% of the standard hFIX activity. For neonatal HB mice, a total AAV mixture of 2 × 10^9^ vg/neonate resulted in sustained hemostatic correction, with the activated partial thromboplastin time (aPTT) values close to those of WT mice. The indel rate in neonatal mice (1.41%) was much lower than that in adult mice (21.55%), potentially due to the rapid dilution of AAV episomes in neonates versus the persistent expression of AAV-Cas9/sgRNA in the adult liver. In addition, most adult mice displayed robust humoral immune responses against the Cas9 protein and AAV2/8, while neonatal mice showed negligible antibody levels against them. Collectively, these results revealed an advantage of applying genome editing at the neonatal stage. When using two or more vectors to deliver the gene editing system, choosing the right vector ratio can further enhance the NHEJ-based KI efficiency. For example, administered at the same total AAV dose, *F8* KO mice receiving AAV8-SaCas9-sgRNA and AAV8-*BDD*-*F8* vectors at a 1:5 ratio gained higher KI efficiency and FVIII activity compared to ratios of 1:2.5 or 1:20 (Chen et al., 2019). With a total AAV dose of 6 × 10^11^ vg/kg (at a 1:5 ratio), the plasma FVIII levels in *F8* KO mice remained above 14% of those in human plasma after 7 months, the bleeding disorder was ameliorated, and no liver toxicity was evident, suggesting a long-term therapeutic potential (Chen et al., 2019).

Alternatively, another homologous recombination (HR)-independent approach, the AAV-trap strategy, was combined with a small Cas9 variant derived from *Campylobacter jejuni* (CjCas9) to mediate targeted expression of the *hF9* gene under the control of the strong promoter of the apolipoprotein C3 (*APOC3*) in hepatocytes (Lee et al., 2022). The AAV-trap vector was designed to encode bidirectional SA and *hF9* sequences, ensuring hFIX protein expression regardless of their direction of integration. With a more complicated NNNNRYAC PAM sequence, CjCas9 represents greater specificity than SpCas9. *In vivo* application of this AAV-trap strategy in F9 mutant mice resulted in sustained reduced clotting time without off-target editing, suggesting a promising new platform for hemophilia therapy (Lee et al., 2022). Subsequently, by combining AAV-trap-mediated *hF9* gene KI with CRISPR/Cas9-mediated antithrombin (AT) gene (Serpin Family C Member 1, *Serpinc1*) disruption for HB therapy, the natural anticoagulation pathway was inhibited, and the endogenous coagulation activity was enhanced at the same time (Lee et al., 2023). A single AAV-donor and lipid nanoparticle (LNP)-CRISPR treatment in HB mice resulted in approximately 1,000 ng/mL plasma hFIX production and inhibition of AT by 66% over a 63-week observation period and successfully restored normal coagulation function (Lee et al., 2023). In a parallel study, combining *Serpinc1* targeting and *BDD-hF8* KI via LNP and AAV vectors also achieved effective and safe gene therapy for HA mice (Han et al., 2023). Of note, the insertion of the *BDD-hF8* gene was mediated by homology-mediated end joining (HMEJ), a highly efficient strategy that incorporated the features of HDR and HITI (i.e., the donor vector harbors sgRNA target sequences and two homology arms) (Yao et al., 2017) (Figure 1B3).

Leveraging the endogenous promoter of the clotting factors has also been investigated to drive therapeutic transgene expression, aiming to restore the expression of the *F8/F9* gene under the control of its native regulatory elements. Ohmori et al. designed a chimeric DNA sequence consisting of a human *F9* SA site and codon-optimized *mF9* cDNA (exons 2–8) with or without homology arms targeting the *mF9* intron 1 (Ohmori et al., 2017). 4–8 weeks post HDR- or NHEJ-mediated AAV8-chimeric *F9* integration, plasma FIX activity in HB mice progressively climbed to 11.7%–39.6%, and a substantial aPTT decrease was observed compared to the non-treated group (Ohmori et al., 2017). A similar approach was applied by targeting the *hF9* cDNA fragment (exons 2–8) containing the *Padua* mutation to the *mF9* exon 2 of *F9* KO mice; thus, a chimeric mRNA was transcribed using the endogenous *mF9* promoter, with the first 117 nucleotides derived from *mF9* and the rest from *hF9co-Padua* (Wang L. et al., 2019). For neonatal *F9* KO mice, the plasma hFIX reached therapeutic levels (10.9% ± 1.6% of normal) 6 weeks post-treatment and exhibited above-normal levels of activity, which remained stable until the study concluded at 32 weeks. The adult *F9* KO mice were subjected to a two-thirds partial hepatectomy 8 weeks post-gene editing but still expressed therapeutic levels of hFIX protein (8.2% ± 1.2% of normal) and possessed above-normal levels of activity (Wang L. et al., 2019). Altogether, these genome-editing strategies are not mutation-position specific and have broad applications to patients with the same disease. In a porcine HB (*pF9* KO) model, site-specific insertion of *hF9* was achieved through CRISPR/Cas9-mediated HDR in the endogenous *pF9* locus, and the FIX activity was elevated to 13.5% compared to 5.5% in the *pF9* KO pigs (Chen J. et al., 2021). The human FIX protein was secreted into the blood of *hF9* KI pigs at a concentration of approximately 90.4 ng/mL, corresponding to 9% of the normal human plasma level, and the bleeding symptoms of hemophilic pigs were greatly alleviated (Chen J. et al., 2021).

Nearly half of the patients with severe HA possess genetic mutations resulting from inversions in introns 1 and 22 of *F8* (Castaman and Matino, 2019). The *F8* intron 1 inversion (Inv1) results from an intrachromosomal homologous recombination, leading to a failure in transcribing *F8* exon 2 and the following exons. Theoretically, reintegrating *F8* exon 1 along with a promoter into intron 1 can restore *F8* expression. Luo and colleagues first established a hemophilia A mouse model by deleting the *F8* promoter region and exon 1, then employed the AAV8-SaCas9 system to reintegrate repair templates into these KO mice (Luo et al., 2021). Two types of donor templates were utilized: type 1 containing a liver-specific promoter, the 146 bp-coding sequences of *mF8* exon 1, and a splice donor site within a homologous sequence targeting intron 1; and the other one, designed by flanking the type 1 donor with two opposing sgRNA sites, is also known as an HMEJ donor. In both cases, ectopic expression of *F8* in hepatocytes (<1%) driven by the human alpha-1-antitrypsin (hAAT) gene promoter was sufficient to shorten the aPTT and improve the survival of HA mice, even though LSECs have been considered the primary source of FVIII production (Luo et al., 2021). This strategy was further explored in induced pluripotent stem cells (iPSCs) derived from HA patients with *F8* Inv1, in which the Inv1 mutation was rescued by the gene addition of the hAAT promoter and *F8* exon 1 (Hu et al., 2023). The donor plasmids were designed for HMEJ-, HDR-, and NHEJ-mediated targeting of *F8* intron 1, with the targeting efficiencies of 10.19%, 6.25%, and 0.99%, respectively, indicating HMEJ is a more efficient gene insertion approach. Furthermore, FVIII expression was restored in hepatocyte-like cells differentiated from gene-targeted iPSCs (Hu et al., 2023).

As a proof of concept, Sarangi et al. employed CRISPR/Cas9 technology to integrate the bypass coagulation factor—mouse activated FVII (mFVIIa)—into the *ROSA26* locus of HB mice to facilitate sustainable clot formation. Eight weeks later, the prothrombin time (PT) assay showed a 22% reduction, and the FVIIa activity was significantly higher in the gene-editing group (122.5 mIU/mL vs. 28.8 mIU/mL in the control). This strategy appears to be versatile and potentially applicable to both HA and HB, as it bypasses the need for FVIII or FIX replacement by directly activating the coagulation cascade with FVIIa (Sarangi et al., 2024).

### 2.2 CRISPR/Cas9-mediated gene repair for hemophilia treatment

*F8* is predominantly expressed in endothelial cells, but the delivery vectors used in current studies are mostly designed to target hepatocytes. This discrepancy limits the application of CRISPR/Cas9-mediated *in situ* gene repair in hemophilia A, whereas its application in hemophilia B has been extensively explored. Guan et al. investigated a treatment for the *F9* Y371D mutation in adult HB mice by HTV injection of a Cas9 plasmid and either a double-stranded plasmid donor template or a short single-stranded oligodeoxynucleotide (ssODN) of 120 nucleotides carrying the edits for HDR gene correction (Guan et al., 2016). The indel and HDR rates were both 0.56% in the ssODN group and were 2.84% and 1.55%, respectively in the plasmid donor group. The average aPTT significantly decreased from 51.88 s in the untreated group to 32.7 s in the ssODN group and 31.6 s in the plasmid donor group. Additionally, the survival rate from the tail-clip challenge significantly increased (from 38% in the untreated group to 86% in the plasmid group), and HTV injections of naked DNA vectors did not cause any histological or functional abnormalities in HB mice. In parallel, recombinant adenoviral (AdV) vectors were applied to deliver the CRISPR/Cas9 system for *in situ* gene repair of pathogenic mutations (Guan et al., 2016). After low-dose and high-dose AdV treatment in HB mice, 0.85% and 5.53% of mutant *F9* alleles were corrected, respectively. Despite higher editing efficiency in the high-dose group compared to that of HTV injection, no significant recovery of coagulation activity was observed, which may be attributed to the high immunogenicity and severe hepatotoxicity induced by AdV. However, this prediction contradicts another study that employed adenovirus-mediated *mF9* cDNA insertion into the *ROSA26* locus and demonstrated long-term therapeutic effects in HB mice (Stephens et al., 2019). Therefore, more studies are needed to optimize this versatile genome editing tool.

High-capacity adenoviral vector (HCAdV), with a high packaging capacity of up to 35 kb, exhibits lower immunogenicity and toxicity compared to early generations of adenoviral vectors, making it an attractive carrier for large genetic payloads in gene therapy. Gao and colleagues employed a single-vector system (HCAdV5-Cas9-donor) to deliver both CRISPR/Cas9 and three different donors (P1, P2, or P3) for HDR of the mutated canine *F9* gene (c.1477G>A; p. G418E) stably expressed in human hepatocellular carcinoma cell lines, which could result in a complete absence of circulating FIX in affected animals (Gao et al., 2019). P1 and P2 featured flanking donor sequences with gRNA recognition sites in either opposite orientation (P1) or in the same orientation (P2), and P3 lacked gRNA recognition sequences entirely. Among these, P1 demonstrated the highest HDR efficiency (5.52%) and the highest secreted cFIX level, highlighting the importance of donor sequence design in optimizing CRISPR/Cas9-mediated gene correction (Gao et al., 2019).

Ohmori et al. utilized AAV8 to deliver the CRISPR/Cas9 system and donor sequence, aiming for *in situ* HDR-mediated gene correction of a 12 bp deletion in the *F9* exon 8 of HB mice (Ohmori et al., 2017). Four weeks after treatment of neonatal and adult HB mice, the plasma FIX activity improved significantly, following a notable enhancement in both aPTT and bleeding phenotypes. To study genetic defects involving promoters/enhancers, Wang et al. first established a severe HB mouse model with mutations covering both the regulatory region upstream of the m*F9* transcription start site and coding regions (Wang et al., 2021). Then a synthetic *Alb* enhancer/promoter-driven *hF9* expression cassette replaced the mutant sequences in HB mice through CRISPR/Cas9-mediated HDR and led to ∼30-fold higher plasma FIX production than that driven by the endogenous m*F9* enhancer/promoter. Additionally, early anti-inflammatory treatment can promote the retention of edited hepatocytes, thereby improving therapeutic outcomes (Wang et al., 2021).

### 2.3 CRISPR/Cas9-mediated inhibition of specific natural anticoagulant processes for hemophilia treatment

Aside from strategies aimed at restoring the function of deficient coagulation factors, enhancing the coagulation capability in hemophilia patients through the inhibition of natural anticoagulation pathways represents another promising approach. Protein S (PS), encoded by the *PROS1* gene, is an anticoagulant that serves as a cofactor for activated protein C and tissue factor pathway inhibitor (TFPI). Studies have demonstrated that knockdown of the *PROS1* in either HA or HB mice can protect them against bleeding, particularly intra-articular hemorrhage (Prince et al., 2018). This is characterized by increased thrombin generation, resistance to activated protein C and TFPI, and an improved fibrin network (Prince et al., 2018). Protein Z (PZ) is a vitamin K-dependent cofactor, while the PZ-dependent protease inhibitor (ZPI) is a plasma protein that collaborates with PZ to inhibit coagulation factor Xa. It has been shown that PZ or ZPI deficiency by gene knockout significantly enhances the coagulation capacity of HA mice (Girard et al., 2019), indicating critical roles of PZ and ZPI in the blood coagulation process and suggesting new therapeutic strategies for hemophilia. Altogether, these are potential genome editing targets with CRISPR/Cas systems.

An RNA interference (RNAi) therapy targeting antithrombin has been demonstrated to promote hemostasis in a hemophilia mouse model and has entered clinical trials (Sehgal et al., 2015). In this context, Ohmori et al. utilized AAV-delivered CRISPR/Cas9 for target disruption of *Serpinc1*, resulting in improved hemostatic ability of HB mice through the inhibition of the natural anticoagulant process (Ohmori et al., 2017). Han et al. managed to achieve a consistent downregulation of *Serpinc1* (up to 70%) in hemophilia mice using LNPs to deliver SpCas9 mRNA and sgRNA, which resulted in enhanced coagulation without off-target effects, liver damage or anti-Cas9 immune responses (Han et al., 2022). As discussed previously, combining *F8/F9* KI with *Serpinc1* disruption further improved coagulation function in hemophilia mice (Han et al., 2023; Lee et al., 2023). Taken together, inhibiting natural anticoagulation pathways through RNAi or CRISPR interference, or CRISPR/Cas9-mediated gene knockout, may be an effective therapeutic approach to alleviate bleeding symptoms in hemophilia patients. The advantages include (1) the treatment remains effective even in patients who have developed inhibitors against clotting factor replacement therapies; (2) gene disruption is more amenable to CRISPR/Cas9 compared to gene insertion, making it better suited for targeting natural anticoagulation pathways; and (3) it is highly suitable for prophylactic treatment. Of note, long-term observation is necessary for any therapy to ensure its sustained efficacy and safety, such as low thrombosis risk and no impact on other physiological functions.

### 2.4 Strategies to overcome CRISPR/Cas9 limitations

Despite the promising prospects of the CRISPR/Cas9 technology for gene therapy, some disadvantages cannot be overlooked. The CRISPR/Cas9 system may pose a risk of off-target effects (Fu et al., 2013), which highly impact the efficacy of gene editing. Several strategies to minimize these effects include screening for high-precision sgRNAs (Wang et al., 2020; Han et al., 2022), optimizing sgRNA design and modifications (Chen X. et al., 2022), employing different Cas9 variants (Guilinger et al., 2014), and utilizing superior delivery methods (Naeem et al., 2020). Employing paired Cas9 nickases can reduce off-target effects because of requiring two nicks to generate a DSB (Shen et al., 2014). However, Cas9 nickases retain catalytic activity and can still induce genomic modifications as monomers. To further enhance specificity, fusing catalytically inactive Cas9 with the inactive catalytic domain of FokI endonuclease creates an fCas9 system. Since FokI is only catalytically active in its dimeric form, only when two fCas9 complexes guided by two independent guide RNAs bind adjacent opposing DNA strands simultaneously do they initiate a double-strand break. This fCas9 system exhibits 140-fold greater specificity than wild-type Cas9 and outperforms the paired Cas9 nickases in respect of off-target activity (Guilinger et al., 2014). Using these methods to mediate the insertion of FVIII or FIX variant genes into the genome may be a favorable choice.

DSBs lead to a high incidence of small insertions, deletions (Cullot et al., 2019), chromosomal translocations (Turchiano et al., 2021), and complex chromosomal rearrangements (Kosicki et al., 2018). These unfavorable and unpredictable repair outcomes largely arise from NHEJ, the primary pathway for DNA damage repair in cells, which operates in an error-prone manner. Whereas HDR-mediated precise gene repair accommodates various types of edits like point mutations, insertions, or deletions, its efficacy is comparatively low (Jeggo, 1998) because HDR is typically active in the G2 and S phases of the cell cycle when homologous DNA fragments are present in the nucleus (Pardo et al., 2009). Hepatocytes in adults are generally in a quiescent state and tend to repair DSBs more efficiently via NHEJ, ensuing random insertions and deletions at the target site. The NHEJ-based HITI strategy allows for the targeted integration of exogenous DNA sequences without the need for a homologous arm and is more efficient than HDR (Suzuki et al., 2016), even though HITI cannot remove pre-existing mutations, such as correcting point or frameshift mutations. As a promising therapeutic gene KI strategy (Yao et al., 2017), the HMEJ faces several limitations, including low efficiency (particularly in dividing cells), restricted vector capacity (homology arm length may exceed the packaging limit of AAV), design complexity (e.g., the need to avoid introducing SAs in homology arms to prevent aberrant splicing), and limited applicability (constrained by delivery vectors for *in vivo* applications), which collectively hinder its broader use in gene editing therapies (Suzuki et al., 2019).

Intercellular linearized Single homology Arm donor mediated intron-Targeting Integration (SATI) is an intronic gene KI method in which a donor template containing a single homology arm and a Cas9 cleavage site is constructed to insert sequences of interest into the target site through non-canonical HDR mediated by a single homology arm or NHEJ machinery (Suzuki et al., 2019). SATI is more flexible and easier for delivery with one homology arm as compared to HMEJ and holds potential for broader applications in gene therapy. Whatever genome editing tools are used, prolonged expression of Cas9 usually increases gene editing efficiency, but prolonged exposure may also raise the indel rate, increase off-target effects, and intensify the immune response to Cas9. Overall, the adverse effects of long-term Cas9 expression outweigh the benefits, and transient expression of Cas9 is preferred. Different PAM sequences can impact the efficiency of CRISPR/Cas9-mediated editing (Zhang et al., 2014), implying the importance of optimal Cas9 variant selection.

Mitigating the immune response to vectors and gene editing components is a crucial issue. Previous studies have identified adaptive immune responses against Cas9 and viral vectors (Stephen et al., 2010; He et al., 2022). Most Cas9 orthologs are derived from *S. aureus* and *S. pyogenes*, both of which have a high prevalence in the human population (Charlesworth et al., 2019). Thus, preexisting adaptive immunity (including both humoral and cellular immunity) to Cas9 proteins has been identified in humans (Charlesworth et al., 2019). Since Cas9 is an intracellular protein, humoral immunity may have limited impact on gene therapy outcomes. However, threats from cytotoxic T lymphocytes against Cas9 raise concern over the destruction of Cas9-expressing cells (Crudele and Chamberlain, 2018). Therefore, tolerance induction or short-term immune suppression with transient expression of Cas9 should be considered (Crudele and Chamberlain, 2018). Whenever feasible, it is advisable to select vectors with reduced inflammatory potential, use intravascular injections, utilize a lower dose, and select tissue-specific promoters. For example, exosomes as a natural vehicle can shield AAV from immune detection, and the exosome-enveloped AAV vectors can effectively evade preexisting neutralizing antibodies. This innovative strategy allows more efficient and prolonged gene delivery, even in patients with prior exposure to AAV vectors, to overcome humoral immunity barriers in gene therapy for hemophilia (Meliani et al., 2017).

In summary, one major advantage of treating hemophilia through transgene insertion is its broad applicability across patients without the need for individualized treatment plans based on specific genetic mutations. Specifically, inserting FVIII or FIX high-activity variants enhances coagulation activity comparatively, which can compensate for low gene targeting rates and minimize high-dose vector-induced toxicity. Future studies should focus on improving KI efficiency and reducing off-target rates. The limited studies on *in situ* correction of gene mutations may be due to the inherent limitations of CRISPR/Cas9 technology and could be expanded with the next-generation gene-editing technologies. Lastly, target-inhibition of natural anticoagulation pathways is also a universal treatment strategy that deserves continued investigations.

## 3 Next-generation gene editing for hemophilia treatment

**FIGURE 2:**
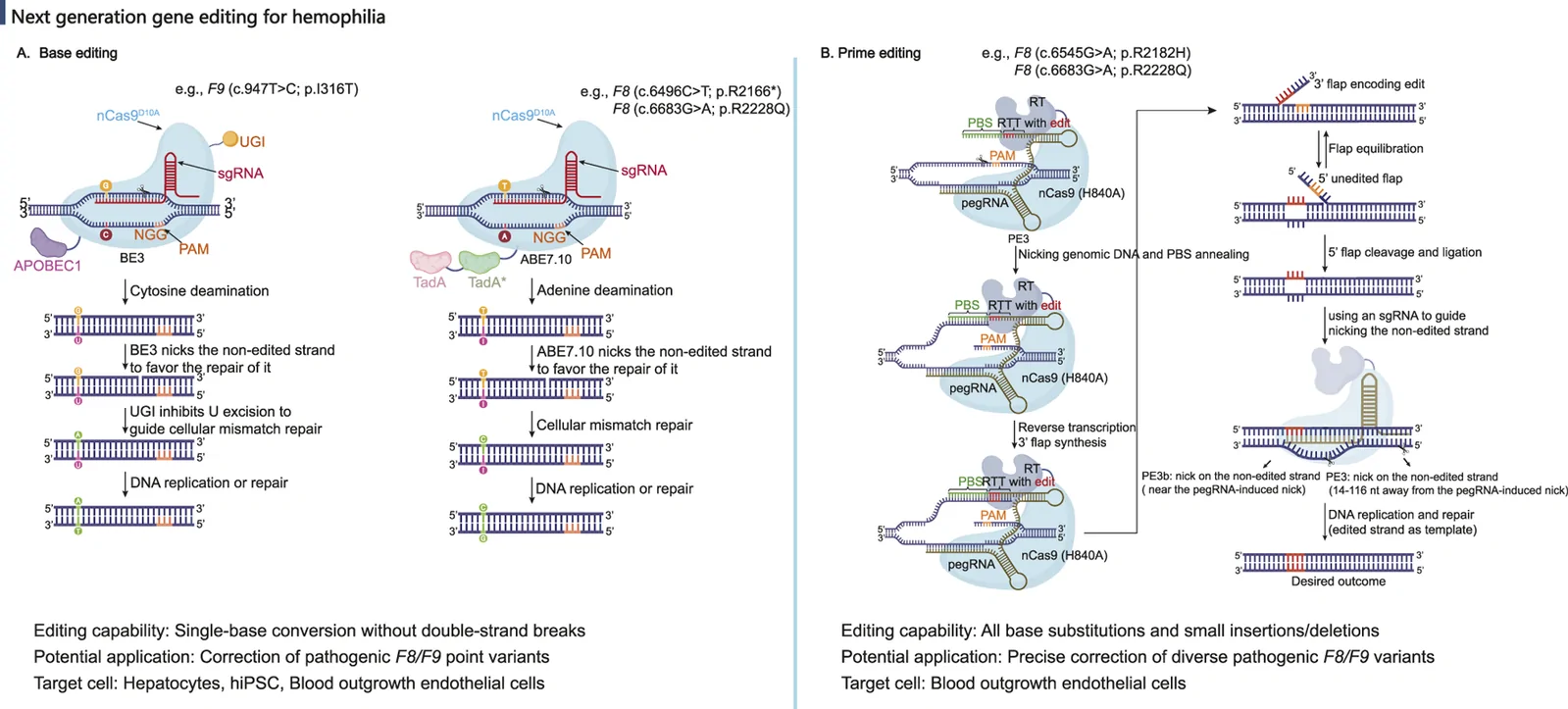
Schematics of base editing and prime editing for the correction of hemophilia. **(A)** A cytosine base editor (CBE, e.g., BE3) consists of nCas9 (D10A), cytidine deaminase (APOBEC1), and uracil glycosylase inhibitor (UGI), enabling C•G to T•A transition at 13–17 nucleotides upstream of the PAM sequence. Adenine base editor (ABE, e.g., ABE7.10) transforms A•T to G•C at 14–17 nucleotides upstream of the PAM sequence. TadA and TadA* are *E. coli* wild-type and evolved adenine deaminases, respectively. **(B)** Prime editors containing nCas9 (H840A), reverse transcriptase domain (RT), and pegRNAs (composed of sgRNA, RT template, and PBS) can achieve all 12 single-base substitutions, insertions, deletions, and their combinations. Both base editors and prime editors mediate gene editing without the need for DSBs or donor DNA templates.

The limitations inherent to the CRISPR/Cas9 system have spurred development of more precise and controllable gene-modifying methods that exhibit high specificity, making edits exclusively at the intended site while minimizing undesired indels. Base editing and prime editing are emerging gene editing techniques that meet these needs, and their potential applications in hemophilia gene therapy are still being explored (Figure 2).

### 3.1 Base editing

At present, base editors (BEs) can be categorized into five types: cytosine base editors (CBEs, transition of C•G to T•A) (Komor et al., 2016), adenine base editors (ABEs, transition of A•T to G•C) (Gaudelli et al., 2017), C•G to G•C base editors (CGBEs) (Chen L. et al., 2021), A•T to C•G base editors (ACBEs) (Chen et al., 2023) and adenine transversion base editors (AYBEs, transversion of A•T to T•A and A•T to C•G) (Tong et al., 2023). Altogether, these base editors can make all kinds of base transitions and transversions possible. Traditional base editors consist of the Cas9 nickase (nCas9, created by replacing key catalytic amino acid residues of Cas9) fused to a deaminase. Guided to the target site by an sgRNA, the nCas9 specifically introduces a single-strand break, followed by adenine or cytosine deamination by a specific deaminase and single base substitution via base excision repair (Komor et al., 2016; Gaudelli et al., 2017; Chen L. et al., 2021). A notable advantage of base editors over the traditional CRISPR/Cas9 system is the avoidance of DSBs, thereby reducing the chances of large deletions and genomic rearrangements and ensuring enhanced precision in genome editing (Komor et al., 2016).

Hiramoto et al. modified CBE by utilizing SpCas9-NG, which recognizes NGN as the PAM sequence, leveraging its broad PAM flexibility to repair pathogenic mutations in hemophilia B (Hiramoto et al., 2023). The *F9* gene point mutation (c.947T > C) was successfully corrected in patient-derived iPSCs and in a knock-in mouse model carrying the same mutation, restoring *F9* gene expression and FIX production. In addition, the efficiency of SpCas9-NG-mediated base editing was around twofold greater than that of wild-type SpCas9, and SpCas9-NG can cover four times as many mutation sites by recognizing only 1 G nucleotide (Hiramoto et al., 2023). More recently, Baatartsogt et al. developed a broadly applicable base editing strategy for HB by introducing a gain-of-function variant into *F9* rather than individually correcting diverse pathogenic mutations (Baatartsogt et al., 2026). The TadCBEd-based cytosine base editor was employed to introduce a G:C-to-A:T substitution at *F9* c.1151, generating the p. R338Q variant, which exhibited threefold higher FIX activity than wild-type FIX. Once delivered with either dual-AAV8 split-intein vectors or LNPs for *in vivo* editing, the TadCBEd system induced around 60% and 25.9%–47.7% on-target editing in mouse liver, respectively, with increased FIX activity. This strategy offers an alternative option for a subset of patients who possess mild or moderate hemophilia B phenotypes (Baatartsogt et al., 2026).

Although base editing has significant potential for therapeutic applications, it still faces technical challenges that limit its clinical translation, including low editing efficiency, bystander editing, and off-target effects (Porto et al., 2020). Base editors typically operate within a small window of 4–5 bases, and if an identical base to the target is present within the activity window, it may also be inadvertently edited, leading to undesired “bystander editing” (Rees and Liu, 2018). Off-target effects are of great concern as well. For instance, CBE3 (BE3) has been shown to induce off-target DNA single nucleotide variants at more than 20-fold higher frequencies compared to ABE7.10 and CRISPR/Cas9 in mouse embryos, which is sgRNA-independent and possibly due to overexpression of the APOBEC1 cytosine deaminase (Zuo et al., 2019). Moreover, both ABE and CBE can induce extensive transcriptome-wide RNA edits, inducing off-target RNA single nucleotide mutations, whereas base editor variants with engineered deaminases show significantly reduced RNA editing activities (Grünewald et al., 2019; Zhou et al., 2019).

Continuous optimizations of base editing systems include introducing new or engineered deaminase domains (Kim et al., 2017; Gehrke et al., 2018; Tan et al., 2019; Jin et al., 2020; Zuo et al., 2020), engineering specific Cas9 variants (Kim et al., 2017; Nishimasu et al., 2018; Huang et al., 2019; Walton et al., 2020), modification of nuclear localization signals (NLS) and codon optimization (Koblan et al., 2018; Zafra et al., 2018), alterations of the linker region connecting the deaminase and Cas9 protein (Komor et al., 2017; Tan et al., 2019; Zhang et al., 2020), and directed evolution (Thuronyi et al., 2019; Lam et al., 2023; Neugebauer et al., 2023). All these efforts aim to enhance editing precision, reduce off-target activity, minimize bystander editing, and expand target compatibility.

### 3.2 Prime editing

Prime editing is an emerging gene editing technology that can achieve all 12 types of single-base substitutions, small insertions or deletions, and their combinations without the need for DSBs or donor DNA templates (Anzalone et al., 2019). The first-generation prime editor (PE1) consists of nCas9 (H840A) fused with a wild-type Moloney Murine Leukemia Virus (MMLV) reverse transcriptase (RT), directed to the target site by the prime editing guide RNA (pegRNA) that carries sgRNA, RT template (with the intended edit), and primer binding site (PBS). Upon binding to the target, nCas9 nicks the PAM-containing strand and releases a 3′ DNA flap that then hybridizes with PBS. The pegRNA-encoded edit was synthesized during the 3′ flap extension and finally incorporated into the genome through the DNA mismatch repair (MMR) mechanism (Anzalone et al., 2019).

The second-generation prime editor (PE2) introduces five mutations in the MMLV RT, allowing for better reverse transcription at elevated temperatures and enhanced pegRNA binding, thus boosting point mutation editing efficiency by 1.6–5.1 times over PE1 (Anzalone et al., 2019). The third-generation prime editor (PE3) introduces an additional sgRNA to guide nicking the unedited strand, promoting the cell’s MMR to favor the edited strand and enhance the retention of the edits. PE3 achieves an editing efficiency of up to 55%, but DSBs increase the chances of indels (Anzalone et al., 2019). This led to the development of PE3b, where the additional sgRNA binds its target sequence only after the pegRNA-directed editing has occurred (Anzalone et al., 2019). PE3b ensures the sgRNA-guided nick is introduced only after the pegRNA-mediated edit has finished, which consequently reduces the indel rate to 0.74%, 13 times lower than that observed with PE3 (Anzalone et al., 2019).

Subsequent studies have indicated that MMR hinders prime editing and leads to the formation of unwanted indel byproducts, which can be reduced through transient expression of a dominant negative MMR protein (MLH1dn) (Chen P. J. et al., 2021). Combining PE2 or PE3 with MLH1dn resulted in the development of PE4 (PE2+MLH1dn) and PE5 (PE3+MLH1dn). Additionally, introducing silent mutations around the editing site can avoid MMR recognition, further increasing editing efficiency (Chen P. J. et al., 2021). Optimizations of the PE protein structure, such as modifying the MMLV RT domain, adding additional NLS, and adjusting the peptide length between Cas9 and MMLV RT, have led to the development of the PEmax framework with enhanced editing efficacy (Chen P. J. et al., 2021). Through phage-assisted evolution and protein engineering, a suite of PE6 variants was developed that demonstrated enhancements in several aspects: PE6a and PE6b achieved reductions in editor size, PE6c and PE6d enhanced RT activity, and PE6e-g improved the Cas9-dependent editing efficiency. For different types of edits, delivery approaches, or specific needs, various PE6 variants were recommended (Doman et al., 2023).

A recent study has shown that the small RNA-binding exonuclease protection factor La played a significant role in prime editing (Yan et al., 2024). Specifically, endogenous La protein engages functionally with the 3′ends of polyuridylated pegRNAs, contributing to the stability and integrity of pegRNAs and engineered pegRNAs. Based on this fact, PE7 was developed by fusing the prime editor protein to the RNA-binding, N-terminal domain of La (Yan et al., 2024). Ongoing optimization of PE7 was carried out in many aspects, such as the linker between the prime editor protein and La, the improved design of La-accessible pegRNAs, and a more condensed PE7 protein with a reduced size (Yan et al., 2024).

Other studies continue to refine prime editing from various aspects. For instance, the integration of structured RNA motifs into the 3′terminus of pegRNAs has been shown to improve stability and protect the 3′extension from degradation, thus increasing editing efficiency without increasing off-target effects (Nelson et al., 2022). Editing efficiency can also be improved by introducing same-sense mutations at proper locations within the RT template of pegRNAs, i.e., pegRNAs containing both the desired edit and additional base substitutions (Li et al., 2022). Additionally, introducing a C/G pair in the small hairpin of pegRNA can stabilize its secondary structure and improve indel efficiency (Li et al., 2022).

The delivery of PE is often hindered by its large size and complex structure. Innovatively, one study separated nCas9 and the RT, creating a split PE system that maintained the precise editing efficiency of the parental unsplit PE with no increase in indel frequency (Liu et al., 2022). Additionally, the split of the pegRNA into sgRNA and a circular RNA RT template also increases editing flexibility. The small size of the split PE system can facilitate its AAV delivery and packaging into lipid nanoparticles (Liu et al., 2022). Achieving large DNA insertions or replacements represented a significant challenge for prime editing, but an increasing number of novel approaches are making this aim possible (Anzalone et al., 2022; Zheng et al., 2023; Sun et al., 2024).

As a proof-of-concept study, Tonetto et al. evaluated the efficacy of BE and PE in correcting HA-causing point mutations, including the rFVIII p. R2166* nonsense mutation and the p. R2228Q, p. R2016W, and p. R2182H missense mutations (Tonetto et al., 2024). The *in vitro* study indicated that the ABE- and PE2-based systems displayed the highest correction effect for the p. R2166* and p. R2228Q variants, respectively. In both cases, the desired A > G conversion efficiency exceeded 24%, corresponding to a 20%–30% recovery of FVIII secretion and activity. Lentiviral delivery of the selected ABE systems into engineered human blood outgrowth endothelial cells resulted in a dose-dependent rescue of functional FVIII secretion. It is noteworthy that the bystander effects of ABE were detected with an efficiency of 38% ± 11%, which may interfere with the therapeutic outcome. Given the variations in editing efficiency and safety across different BE/gRNA and PE/pegRNA combinations, it is essential to screen multiple options to select those that can provide effective editing and minimal off-target effects, ensuring an optimal balance between editing efficiency and safety.

Through genome-wide analyses, Fiumara et al. revealed that both BEs and PEs induced DSBs and genotoxic byproducts, such as deletions and translocations in human hematopoietic stem cells, albeit at a frequency lower than that observed with Cas9 (Fiumara et al., 2023). When utilizing advanced generations of PEs, it is essential to ascertain whether this issue has been ameliorated, and the long-term safety and efficacy of prime editing should be validated before proceeding with clinical trials.

## 4 AAV vectors and next-generation delivery technologies

**FIGURE 3:**
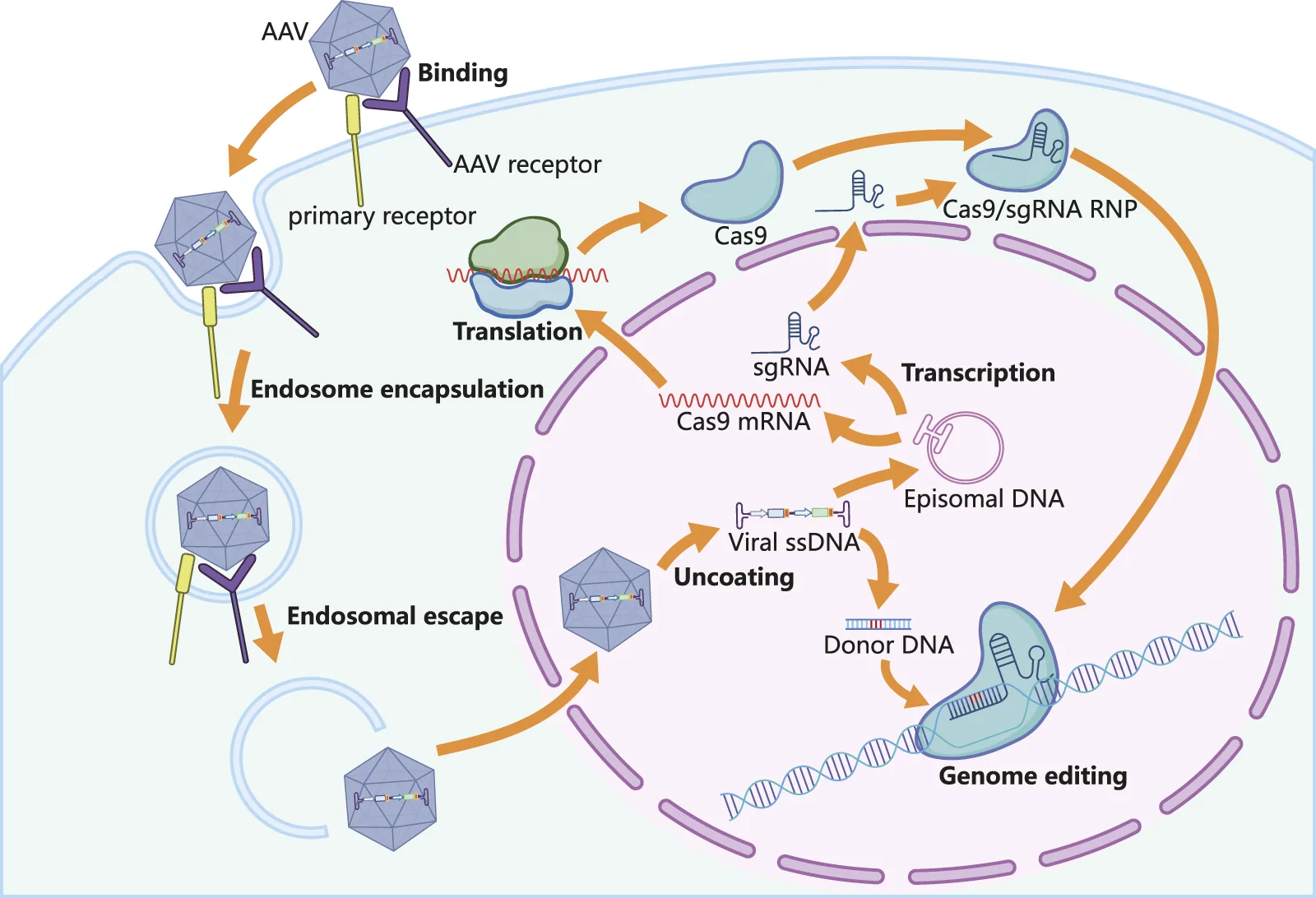
Schematic illustration of AAV-mediated CRISPR/Cas9 genome editing. Following cellular binding and endocytosis, the AAV vector is encapsulated within endosomes and subsequently escapes into the cytoplasm. After uncoating, the viral genome is released and undergoes transcription and translation to produce Cas9 protein and sgRNA. The assembled Cas9-sgRNA complex then locates the target genomic site and induces site-specific DNA cleavage, enabling genome editing.

An optimal delivery system would offer a generous packaging capacity, minimal to no immunogenicity or toxicity, and the ability to deliver the gene editing system efficiently and safely to target cells. AAV vectors (Figure 3), lipid nanoparticles (LNPs), virus-like particles (VLPs), and exosomes all possess the potential to meet these criteria. Combined with next-generation gene editing technologies, these delivery methods could further enhance therapeutic outcomes (Supplementary Figure S2).

### 4.1 Adeno-associated virus

AAV is a non-enveloped, single-stranded DNA virus, and its genome is packaged in an icosahedral capsid of protein shell assembled with 60 subunits of structural viral proteins of VP1, VP2, and VP3. The capsid measures approximately 25 nm in diameter with a genome size of 4.7 kb (Pupo et al., 2022). Recombinant AAVs are generated by replacing their native replication (rep) and capsid (cap) genes from wild-type AAVs with a custom-built gene cassette (therapeutic transgene sequences) flanked by the retained inverted terminal repeats (ITRs), which are crucial for guiding AAV genome replication and packaging during vector production. This design prevents the virus from multiplying inside the patients and significantly reduces the risk of genomic integration and oncogenesis. Meanwhile, rAAVs exhibit broad tissue tropisms of various serotypes determined by the specific composition and structure of their capsids, allowing them to infect both dividing and non-dividing cells across a wide variety of human organs. These features make rAAVs currently the most widely used delivery vectors in gene editing therapies due to their low immunogenicity and pathogenicity, high biocompatibility, and stable transgene expression.

As mentioned before, the Food and Drug Administration in the United States (FDA) approved three AAV-based gene therapies for hemophilia A and B by one-time intravenous infusion: Hemgenix (2022) and Beqvez (2024) for hemophilia B and Roctavian (2023) for hemophilia A. As of August 2026, there are 38 clinical trials involving AAV-mediated gene therapy for hemophilia registered on the ClinicalTrials.gov website. In contrast, only one phase I/II AAV-based CRISPR/Cas9 clinical trial for hemophilia is ongoing, aiming for targeted insertion of the human FIX gene to treat severe or moderately severe hemophilia B (Regeneron Pharmaceuticals, ClinicalTrials.gov ID: NCT06379789).

Despite the widespread use of rAAVs in gene therapy, several limitations and challenges still need to be addressed. It has been shown that the dose of AAV vectors used is closely linked to the severity of immune responses and associated adverse events (Ertl, 2022). To overcome the loading capacity of AAV vectors, a dual-AAV approach is commonly used to deliver CRISPR/Cas9 components (Ohmori et al., 2017; Chen et al., 2019; Wang L. et al., 2019; Wang et al., 2020), which significantly increases the AAV injection dose and may lead to more potent immune reactions and associated side effects. High doses could increase the risk of off-target integrations (Barzel et al., 2015). A more compact gene editor delivered by a single AAV vector might address this issue. Typically, rAAVs express therapeutic transgenes persistently, which is essential for gene augmentation therapy. However, prolonged expression of gene editing agents also increases the risk of off-target edits (Anzalone et al., 2020), and elicits an immune response in the body, including humoral and cellular immunity, which can potentially lead to the clearance of edited cells (Chew, 2018). Thus, minimizing the duration of gene expression holds significant importance.

AAV-delivered CRISPR systems universally exhibit genotoxic potential through vector fragment integration at DNA break sites, irrespective of Cas9 variant, tissue type, or target gene. Key findings reveal: (1) AAV integration dominates editing outcomes (47%–89% of modifications), comprising heterogeneous fragments including complete cassettes, truncated segments, or isolated ITRs; (2) integrated vector elements (promoters, polyA signals, and Cas9 coding sequences) persistently disrupt endogenous gene function and pose oncogenic risks; (3) integration occurs exclusively at on-target breaks without elevating genome-wide background insertion (Hanlon et al., 2019; Bazick et al., 2024). These risks’ severity can be modulated through: (1) Vector design: minimizing cassette size, engineering ITRs to reduce DNA fusion affinity, and employing tissue-specific promoters to limit Cas9 expression; (2) gRNA strategy: Prioritizing single gRNA designs to reduce double-strand breaks, with multi-targeting approaches requiring careful risk-benefit analysis; (3) Clinical protocols: Avoiding NHEJ inhibitors that exacerbate integration while implementing AMP-seq for preclinical insertion monitoring. Overall, AAV integration is a major gene-editing consequence and must be carefully monitored during its applications.

AAVs are ubiquitous in nature, and some individuals have preexisting Nabs against AAVs. Depending on geographical location, the prevalence of anti-AAV2 Nabs in populations ranges from 30% to 60%, whereas anti-AAV1, AAV7, AAV8, and AAV9 range from 15% to 30% (Vandamme et al., 2017). Previous studies have shown that even low levels of Nabs are sufficient to block AAV transduction in the liver (Hurlbut et al., 2010). Moreover, anti-AAV Nabs against serotypes with highly conserved capsid sequences (like AAV1 and AAV2) can cross-neutralize serotypes with similar capsid sequences (Majowicz et al., 2019). As a result, patients with these antibodies are often excluded from AAV-based hemophilia gene therapy clinical trials (Wang D. et al., 2019). For patients lacking Nabs in the body, the vector capsid elicits strong humoral immunity following rAAV injection, rendering a second dose typically unfeasible. To facilitate repeated administrations, the induction of immune tolerance may be necessary (Wang D. et al., 2019). Besides adaptive immune responses, innate immunity against AAV could also pose significant risks. Recently, a clinical trial using rAAV9-CRISPR to treat Duchenne muscular dystrophy reported a fatality in which an intense innate immune response triggered by a high dose of rAAVs led to acute respiratory distress syndrome, ultimately causing death (Lek et al., 2023). Thus, developing alternative delivery systems might alleviate the existing challenges with rAAV vectors and promote further development in gene editing therapies for hemophilia.

### 4.2 Lipid nanoparticle

LNPs are artificially synthesized and typically composed of ionizable cationic lipids, zwitterionic phospholipids, polyethylene glycol lipids, and cholesterol (Kazemian et al., 2022). LNPs exhibit remarkable affinity for hepatocytes. Upon intravenous administration, apolipoprotein E **(**ApoE) attaches to the surface of LNPs, leading to internalization of LNPs into hepatocytes through endocytosis triggered by the interaction of ApoEs with surface lipoprotein receptors (Akinc et al., 2019). LNPs possess significantly lower immunogenicity compared to viruses and in some cases can be readministered (Kenjo et al., 2021; Han et al., 2022). The Cas9 mRNA and sgRNA delivered by LNPs can be rapidly degraded *in vivo* (Finn et al., 2018), which can significantly decrease the chances of off-target editing and the risk of immune responses induced by the Cas9 protein. Unlike rAAV vectors, LNPs are not restricted by packaging size or preexisting immunity (Han et al., 2022) and do not mediate gene integration events by delivering mRNA or ribonucleoprotein (RNP).

A recent study applied an ionizable cationic lipid MC3-based LNP to deliver Cas9 mRNA and sgRNAs targeting the mutant *F8* gene in an HA mouse model, which carried a 5-base pair deletion in *F8* exon 1, causing a premature stop codon (Chen et al., 2024). Indel variants generated at the mutant site mainly contained 1-base pair and 4-base pair deletions, resulting in frameshift correction of the *F8* gene. The average gene editing rates in hepatocytes and LSECs were 60.54% and 16.50%, respectively. The treated mice could maintain FVIII activity of 3.30% ± 0.68% over a period of 26 weeks and showed improved coagulation.

Zhao et al. designed a biomembrane-inspired LNP system, comprising sphingomyelin and C18-galactosyl ceramide, to enhance liver-targeted CRISPR delivery efficiency (Zhao et al., 2025). The study employed a sequential treatment strategy: the AAV8 vector encoding the *BDD-F8* repair gene was administered first, followed by LNP-mediated delivery of Cas9 mRNA and sgRNA (targeting the *Alb* intron 13) 2 weeks later, achieving site-specific genomic integration of the *BDD-F8* cassette at the albumin locus. A single dose of LNP successfully restored plasma FVIII activity to more than 50% of wild-type levels for over 12 weeks in HA mice without off-target editing or significant toxicity and systemic cytokine induction.

It is essential to improve therapeutic efficacy by introducing appropriate chemical modifications to the sgRNA, thus reducing its susceptibility to nuclease degradation and increasing its encapsulation efficiency (Yin et al., 2017). Additionally, LNP composition and formulation have great impacts on its efficacy (Padilla et al., 2025). In a clinical trial using LNPs to encapsulate Cas9 mRNA and sgRNA for the treatment of transthyretin amyloidosis (Gillmore et al., 2021), almost no adverse events occurred, validating the safety of LNPs in human applications. In addition, repeated LNP-CRISPR injections elicited much less immune response than the persistent AAV-Cas9 expression in hemophilia mice (Han et al., 2022). Utilizing anionic lipid nanoparticles as a delivery system can preferentially deliver mRNA to the hepatic reticuloendothelial system (Pattipeiluhu et al., 2022), compensating for the usual inability of viral vectors to target hepatic sinusoidal endothelial cells, presenting new hope and opportunities for hemophilia A treatment. A clinical trial using a dual-vector system combining LNP and AAV is undertaken recently with LNP-encapsulated Cas9 mRNA and a single guide RNA (sgRNA) targeting liver cells to insert FIX (NCT06379789).

### 4.3 Virus-like particle

VLPs are self-assembling structures containing viral proteins essential for efficient and tissue-specific targeting but lacking the viral genetic material and are therefore non-infectious. Viral capsid proteins can package nucleases in the form of mRNA, protein, or RNP and the resulting VLPs can be applied for genome editing (Lyu et al., 2020). Like LNPs, VLPs offer transient expressions of the gene editing system, substantially reducing the side effects of prolonged expression. VLPs enter target cells via receptor-mediated endocytosis and can escape from endosomes before lysosomal degradation, making them highly suitable for delivering gene editing systems (Nooraei et al., 2021). Some VLPs exhibit the same tropism as the viruses from which they are derived (Nooraei et al., 2021). For instance, VLPs derived from the hepatitis B virus naturally infect liver cells, making them appropriate for diseases like hemophilia.

Liu and colleagues developed the fourth generation (V4) engineered DNA-free VLPs (eVLPs) through extensive modification of the protease-cleavable linker sequence, gag–cargo localization, and gag–cargo:gag–pro–pol stoichiometry (Banskota et al., 2022). *In vitro* and *in vivo* experiments indicated that V4 eVLPs combined the advantages of both viral and non-viral delivery strategies, including high efficiency, tissue specificity, transient delivery, minimized off-target editing, and reduced risk of DNA integration (Banskota et al., 2022). A single intravenous injection of V4 eVLPs to deliver adenine base editor RNPs targeting proprotein convertase subtilisin/kexin type 9 (*Pcsk9*) in mouse liver achieved 63% editing efficiency, comparable to that obtained with either AAV-based or LNP-based mRNA delivery systems without off-target editing (Banskota et al., 2022). Recently, a modified CBE system, transformer base editor (tBE), was packaged in VLPs, and a single injection of tBE-VLP4 achieved 46.0% editing at *mPcsk9* in mouse liver with no detectable off-target edits (Zhu et al., 2026). With an iterative process, an all-in-one eVLP delivering prime editor RNPs was developed, and a single dose of v3 PE-eVLPs demonstrated around 7.2% and 15% editing efficiency for correcting a nonsense mutation in *Rpe65* (retinal pigment epithelium 65) and a 4-bp deletion in *Mfrp* (membrane-type frizzled-related protein) genes, respectively, in mouse models (An et al., 2024). Han et al. further expanded the targeting capabilities and tailored the tropisms of VLPs through engineering the envelope proteins via rational peptide insertion or pseudotyping (Han et al., 2025). Similar to most viral vectors, VLPs induce the production of neutralizing antibodies, which often make subsequent administrations ineffective and necessitate further investigation (Han et al., 2025).

### 4.4 Exosome

Exosomes are a type of extracellular vesicle (EV) with a size of 40–160 nm and are formed through the inward budding of a multivesicular body (MVB) after the fusion of MVB and the plasma membrane (van Niel et al., 2018; Chen T. Y. et al., 2022). Serving as a cellular communication mechanism, EVs can transfer components such as proteins, lipids, and nucleic acids, thereby influencing both the physiological and pathological functions of parent and recipient cells (Yáñez-Mó et al., 2015). Exosomes offer inherent biocompatibility, efficient transport capacity, stability in the bloodstream, and the potential for engineering. Moreover, compared to other non-viral vectors, exosomes exhibit reduced immunogenicity and minimal toxicity (Wan et al., 2022). The gene editing system can be encapsulated within exosomes in the form of RNA or RNP to mediate *in vivo* gene editing, such as loading Cas9 RNPs into purified exosomes isolated from hepatic stellate cells through electroporation (Wan et al., 2022). Post-systemic administration of exosome^RNP^ in healthy mice demonstrated the biodistribution of exosomes across different liver cell types, with the exosome-positive rate of 58.2% and 22.9% in LSECs and hepatocytes, respectively (Wan et al., 2022). In addition, exosome^RNP^-mediated genome editing showed robust therapeutic effects in acute liver injury, chronic liver fibrosis, and hepatocellular carcinoma mouse models (Wan et al., 2022), indicating the potential of exosomes as powerful delivery platforms for gene editing treatments for both HA and HB.

## 5 Discussion and future perspectives

According to statistics from the U.S. Centers for Disease Control and Prevention (CDC) Hemophilia Mutation Project (CHAMP and CHBMP), point mutations account for more than half of the cases in both HA and HB patients, even in severe cases. Therefore, using base editing or prime editing to precisely correct point mutation *in situ* is a feasible treatment option that induces less genotoxicity than Cas9. Moreover, prime editing can introduce small indels, which could benefit even more hemophilia patients.

One concern in hemophilia gene therapy is the episomal expression of the transgene in host cells, which dilutes the transgene through cell division. LSECs and hepatocytes originate from different embryonic germ layers, with LSECs from the mesoderm, whereas hepatocytes are from the endoderm. The classic paradigm of hepatocyte regeneration dictates that liver cell turnover is incredibly slow during homeostasis over the human lifespan and primarily relies on the self-duplication of mature hepatocytes rather than a distinct classical stem cell pool. A landmark study by Dr. Olaf Bergmann’s team proved that the human liver remains a young organ. Diploid hepatocytes divide at a rate of 19% per year and have an average age of 2.7 years in young adults (25 years old) compared to a 17% dividing rate and an average age of 2.9 years in middle-aged and old individuals (50–75 years old), while liver non-hepatocytes demonstrate an average dividing rate of 16% in young and 13% in old individuals (Heinke et al., 2022). Therefore, theoretically desired gene editing or transgene expressions in hepatocytes or LSECs can be partially passed on to daughter cells through cell division, as happens in progenitor cells.

Although AAV-CRISPR/Cas9 could implement the targeted integration and correct defective gene functions in primary human hepatocytes derived from patient livers and expanded with special hepatocyte medium (Zhang et al., 2024), isolation and *ex vivo* gene editing of autologous hepatocytes remains a big challenge technically and practically. Liver-targeted insertion of *F8* or *F9* transgenes by CRISPR systems remains an ideal strategy to achieve long-term efficacy. Recombinant AAV vectors are the primary delivery system, with AAV5 and AAV8 being the most used serotypes in clinical trials for low pre-existing antibodies (AAV5) and high transduction efficiency (AAV8) in human hepatocytes and sinusoidal endothelial cells. Owing to the AAV packaging size limit, dual vectors such as AAV and LNP may be required to distribute viral components across two separate vectors. In addition, switching from AAV to lentiviral vectors could be an alternative. Younger pediatric patients may also benefit from this switch because genomic integration ensures persistent long-term transgene expression, overcoming the dilution of episomal AAV vectors during their rapid liver growth and offering a potential lifetime correction. The lentiviral vector is also a suitable candidate for patients with pre-existing antibodies to AAV capsids in gene therapy.

Alternatively, both autologous and heterologous cell therapies have emerged as potential treatment strategies for hemophilia. Several studies applied gene editing techniques to integrate therapeutic transgenes into the genome of hemophilia patient-derived iPSCs, which were then differentiated into cells that can produce FVIII or FIX. Upon transplantation into hemophilic mice, these gene-edited cells can alleviate the symptoms of hemophilia (Luce et al., 2022; Kim et al., 2025). Another innovative strategy worth noting is the development of autologous B cell therapy through gene editing (BE-101), in which CRISPR/Cas9 was used to mediate site-specific insertion of the *F9* transgene in B cells (Liu et al., 2025). Upon BE-101 engraftment in mice, steady circular FIX Padua secretion was detected for over 184 days without adverse effects. This autologous cell-based therapeutic offers advantages such as no need for host preconditioning, repeatable dosing and dose adjustability, providing a new treatment option for patients with hemophilia.

With continuous advancements in gene editing, vector delivery, and other related technologies, we are stepping towards achieving the goal of efficient, precise, and low-side-effect gene editing therapies. For example, to reduce genotoxicity and off-target cleavage, high-fidelity editors such as *eSpCas9* and *SpCas9-HF1* and the incorporation of a self-inactivating system can be applied, allowing the CRISPR machinery to cut its own encoding plasmid or vector once the target genome edit is complete. Liver resident macrophages, Kupffer cells, internalize and clear a massive portion of the systemic AAV payload from circulation before it can reach hepatocytes; hence, engineering AAVs with CD47 signaling peptides, which bind to macrophages and inhibit their phagocytosis, may be a prerequisite. Whatever strategies are used, ongoing safety tracking is required to monitor liver health, potential AAV integration and genomic interactions.

Lastly, the objective of gene editing therapy is a lifelong cure with just one treatment. As we strive to optimize gene editing results through diverse techniques, it is crucial to continually assess the longevity of therapeutic effects. Continuous monitoring of current and future research outcomes is paramount to ensuring the long-term efficacy and safety of these treatments.
