# Supplementary material for: Advancements in CRISPR-based in vivo gene therapy for hemophilia
Source: Front Genome Ed. 2026 Sep 10;8:1924733. doi: 10.3389/fgeed.2026.1924733 (PMC13601842; doi:10.3389/fgeed.2026.1924733)
Supplement: Supplementary file 1 [file DataSheet1.pdf]

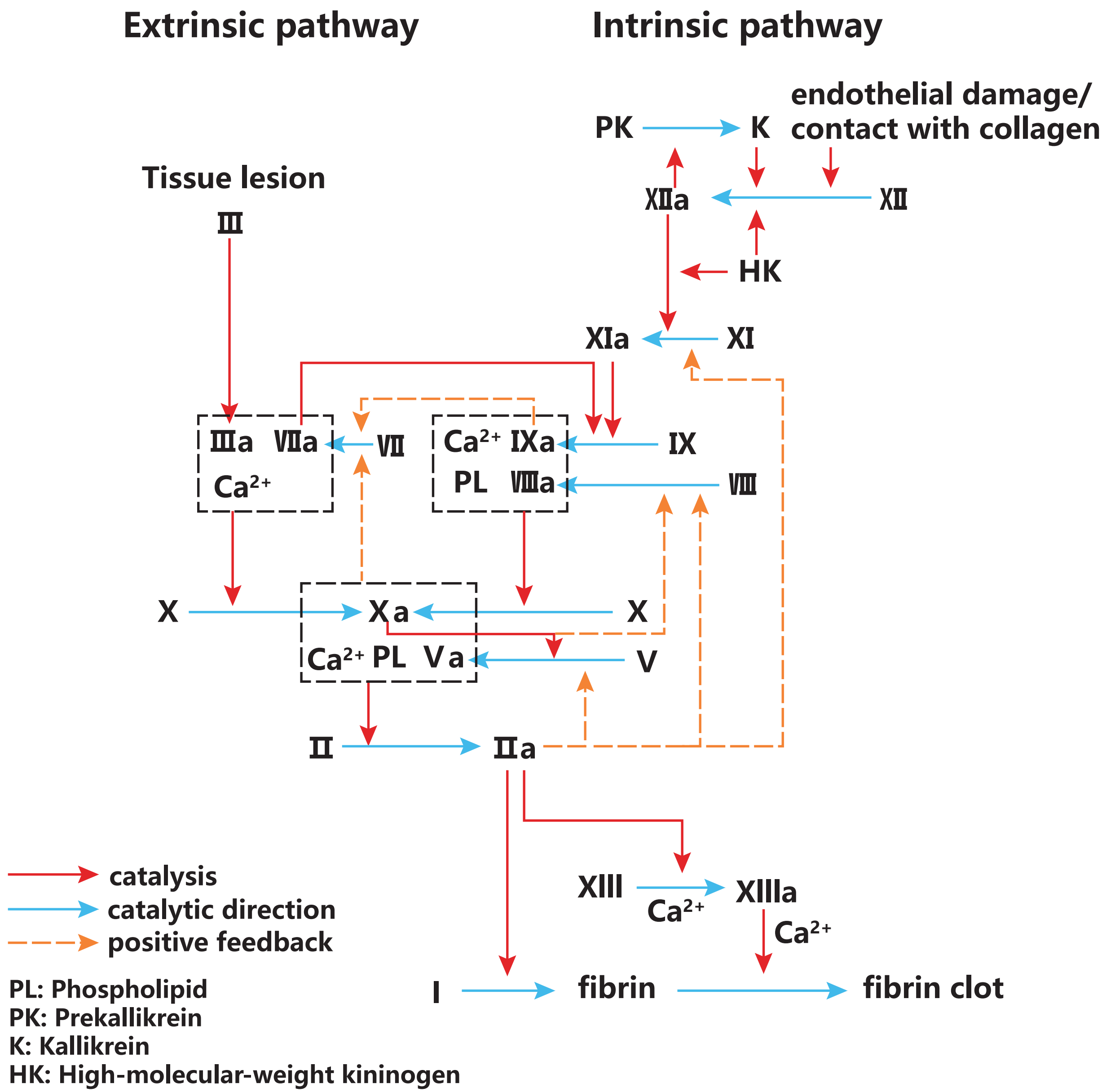
Extrinsic pathway
Intrinsic pathway
endothelial damage/
contact with collagen
PK
K
Tissue lesion
III
XIIa
XII
HK
XIa
XI
IIIa VIIa
Ca2+
VII
Ca2+ IXa
PL VIIIa
IX
VIII
X
Xa
X
Ca2+ PL Va
V
II
IIa
XIII
Ca2+
XIIIa
Ca2+
I
fibrin
fibrin clot
catalysis
catalytic direction
positive feedback
PL: Phospholipid
PK: Prekallikrein
K: Kallikrein
HK: High-molecular-weight kininogen

Supplementary Figure 1. Schematic of coagulation cascade. The coagulation cascade is a tightly regulated sequence of enzymatic reactions that occur in response to tissue injury or endothelial damage, forming the core of secondary hemostasis.
