# Supplementary material for: Advancements in CRISPR-based in vivo gene therapy for hemophilia
Source: Front Genome Ed. 2026 Sep 10;8:1924733. doi: 10.3389/fgeed.2026.1924733 (PMC13601842; doi:10.3389/fgeed.2026.1924733)
Supplement: Supplementary file 2 [file DataSheet2.pdf]

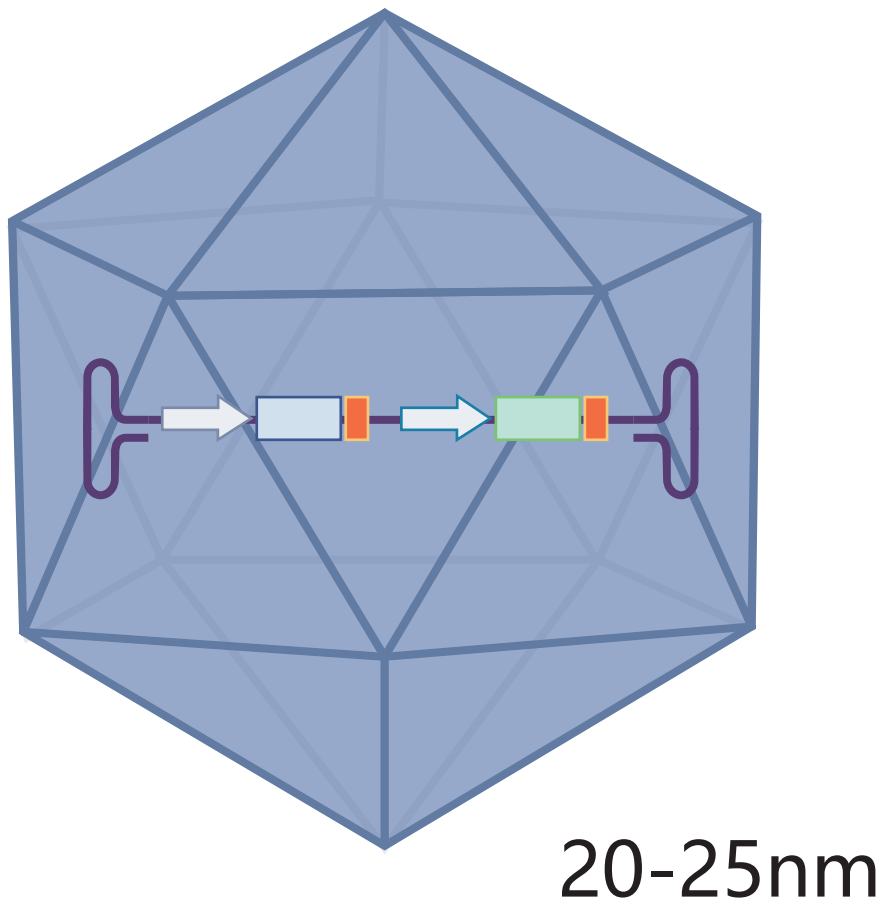

Name : Adeno-associated virus

Form of gene editing agents : DNA

Advantages :
- low immunogenicity and pathogenicity
- high biocompatibility
- broad tissue tropism with various serotypes

Name : Lipid nanoparticle

Form of gene editing agents : mRNA and RNP

Advantages :
- lower immunogenicity compared to viruses
- high biocompatibility
- transient expression and no Cas9 DNA integration
- not restricted by packaging size or preexisting immunity

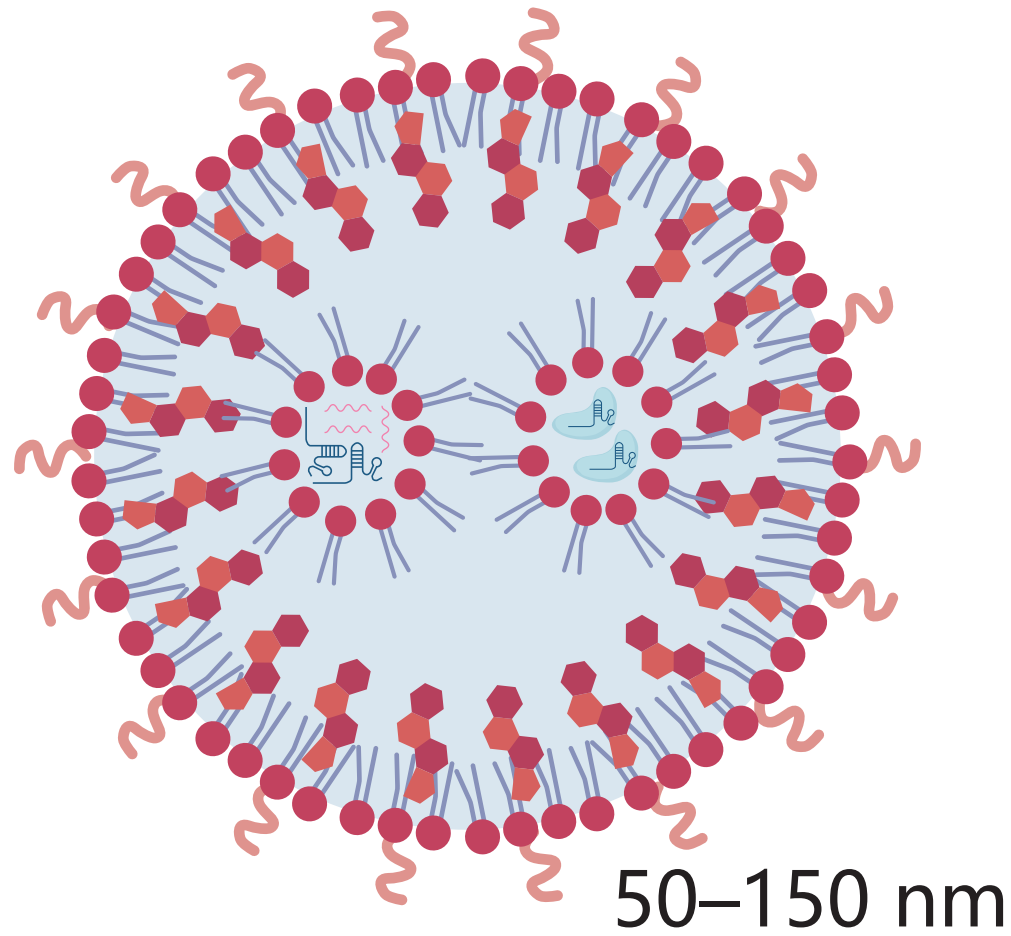

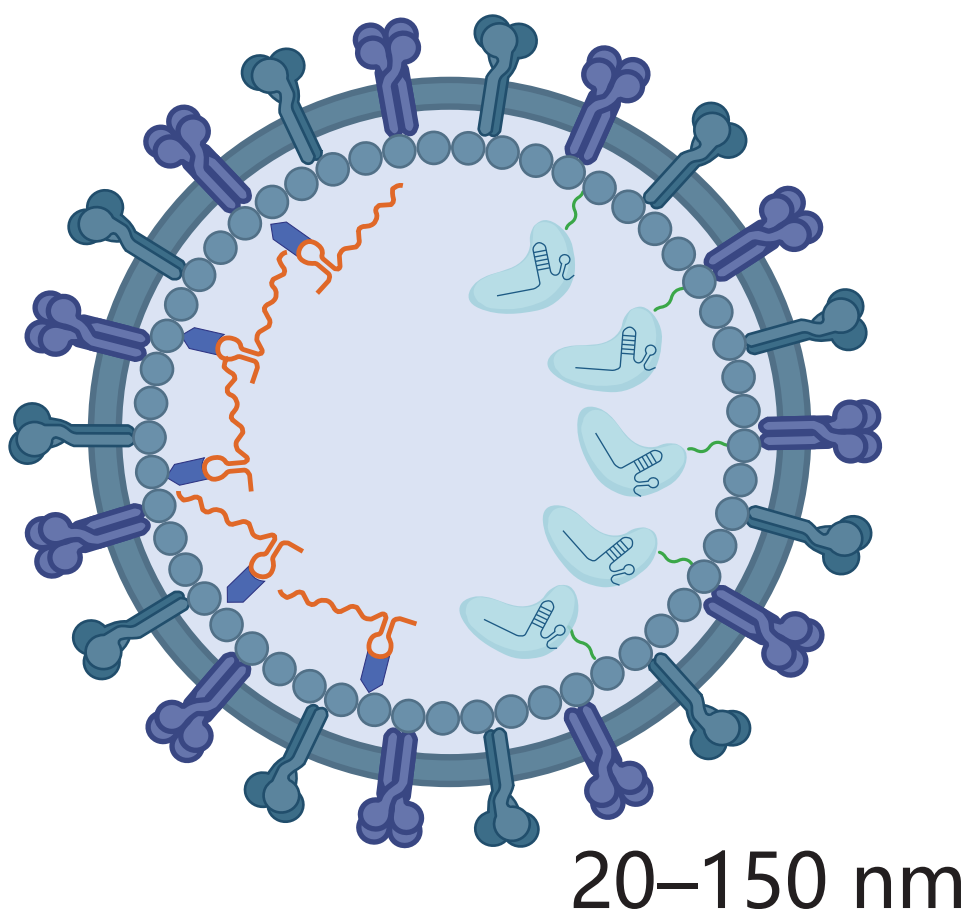

Name : Virus-like particle

Form of gene editing agents : mRNA and RNP

Advantages :
- combining advantages of both viral and non-viral vectors
- endosomal escape before lysosomal degradation

Name : Exosome

Form of gene editing agents : plasmid DNA, mRNA and RNP

Advantages :
- inherent biocompatibility
- efficient transport capacity
- stability in the bloodstream
- reduced immunogenicity and minimal toxicity

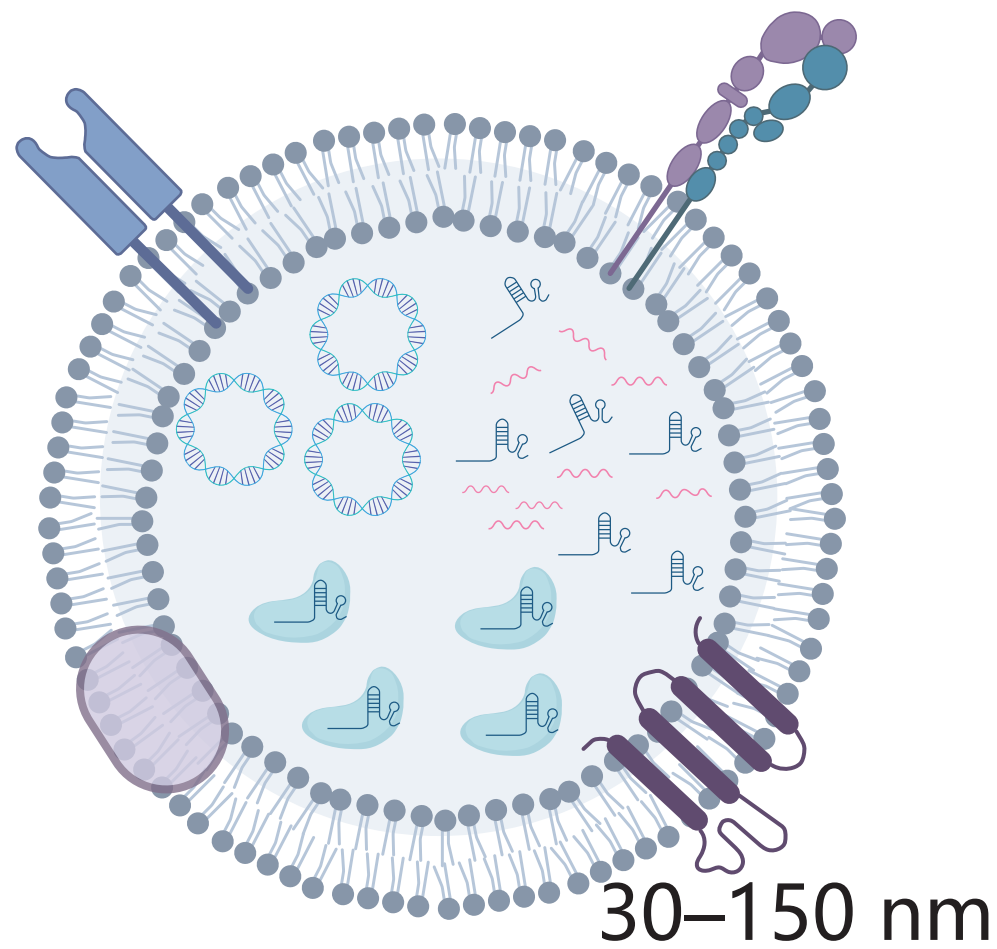

Supplementary Figure 2. Delivery systems for gene editing agents. Comparison of AAV, LNP, VLP, and exosomes as delivery systems in terms of the gene editing agents they can deliver and advantages of each tool.
